# Variables and Strategies in Development of Therapeutic Post-Transcriptional Gene Silencing Agents

**DOI:** 10.1155/2011/531380

**Published:** 2011-06-30

**Authors:** Jack M. Sullivan, Edwin H. Yau, Tiffany A. Kolniak, Lowell G. Sheflin, R. Thomas Taggart, Heba E. Abdelmaksoud

**Affiliations:** ^1^Department of Ophthalmology, University at Buffalo SUNY, Buffalo, NY 14214, USA; ^2^Department of Pharmacology and Toxicology, University at Buffalo SUNY, Buffalo, NY 14214, USA; ^3^Department of Physiology and Biophysics, University at Buffalo SUNY, Buffalo, NY 14214, USA; ^4^Neuroscience Program, University at Buffalo SUNY, Buffalo, NY 14214, USA; ^5^Ross Eye Institute, University at Buffalo SUNY, Buffalo, NY 14209, USA; ^6^Veterans Administration Western New York Healthcare System, Medical Research, Buffalo, NY 14215, USA; ^7^Department of Neuroscience and Physiology, Upstate Medical University, Syracuse, NY 13215, USA

## Abstract

Post-transcriptional gene silencing (PTGS) agents such as ribozymes, RNAi and antisense have substantial potential for gene therapy of human retinal degenerations. These technologies are used to knockdown a specific target RNA and its cognate protein. The disease target mRNA may be a mutant mRNA causing an autosomal dominant retinal degeneration or a normal mRNA that is overexpressed in certain diseases. All PTGS technologies depend upon the initial critical annealing event of the PTGS ligand to the target RNA. This event requires that the PTGS agent is in a conformational state able to support hybridization and that the target have a large and accessible single-stranded platform to allow rapid annealing, although such platforms are rare. We address the biocomplexity that currently limits PTGS therapeutic development with particular emphasis on biophysical variables that influence cellular performance. We address the different strategies that can be used for development of PTGS agents intended for therapeutic translation. These issues apply generally to the development of PTGS agents for retinal, ocular, or systemic diseases. This review should assist the interested reader to rapidly appreciate critical variables in PTGS development and facilitate initial design and testing of such agents against new targets of clinical interest.

## 1. PTGS Technologies

The basic mechanisms of antisense (AS), ribozyme (Rz), and RNA interference (RNAi) approaches to PTGS will be presented here. A comparison of their properties is presented (Table 1). 

### 1.1. Antisense

**Figure 1:**
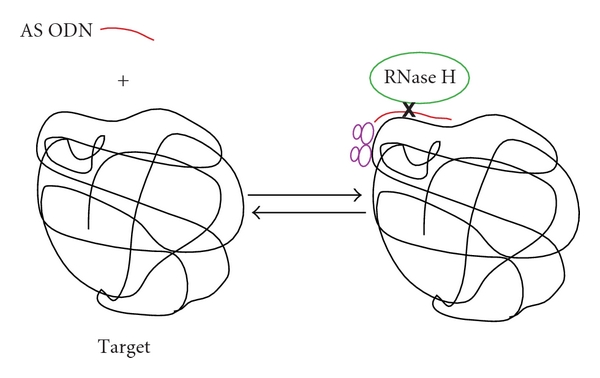
Antisense mechanism. A schematic representation is shown for two dominant mechanisms by which AS ODN molecules delivered into cells can suppress gene expression. The AS ODN must first anneal to an accessible region of the target mRNA. The first and likely dominant mechanism of inhibition is through recruitment of RNaseH (green) to cleave the RNA is the center of the ODN: Target RNA hybrid region. The second mechanism involves physical hindrance of biochemical processes operative on the mRNA such as ribosome- (violet-) mediated translation, 5′ decapping, and 3′ deadenylation. Here, the hybridized ODN is depicted blocking the progress of translating ribosomes on the mRNA.

 AS intended for clinical use is an oligodeoxynucleotide (ODN) string with bases chosen to form Watson Crick annealing pairs over an accessible region of the target mRNA or viral RNA. Various backbone formulations have been used with the intent of resisting nuclease degradation outside or inside cells, enhancing, the binding energy to the target RNA, reducing the strong electrostatic repulsive energies during annealing, and enhancing specificity of RNase H attack. Modifications to the intrinsic phosphodiester backbone chemistry include: phosphorothioate, methylphosphonoester, peptide nucleic acid, 2-ortho-methyl-deoxyribose, locked nucleic acid, and morpholino. Chemical modifications influence cellular uptake, and AS ODNs are provided to tissues directly rather than being expressed within cells from a genetic construct. Chemical modifications of ODNs and such engineered properties are not the focus here, and an interested reader should consult prior literature [1–4]. Single-stranded ODNs are transfected or transduced into cells where they diffuse and encounter target RNAs in either the nucleus or cytoplasm. Two generally accepted mechanisms of AS ODN inhibition of gene expression are both dependent upon strong annealing to the target RNA (Figure 1). These include ODN catalysis of target RNA degradation by RNaseH and/or physical blockade mechanisms (e.g., translation block through ribosome stalling, blocking splicing, blocking polyadenylation) [1, 2, 4, 5]. The first mechanism can occur anywhere in the processed RNA, whereas the second mechanism must occur within the coding region of the target or at sites of splicing or polyadenylation. The translating ribosome can remove antisense ODNs due to its helicase function [6]. Therefore, AS conformational block may best be conducted at or in proximity of the translation initiation codon. AS inhibition of target gene expression can, therefore, occur at the post-transcriptional or cotranslational levels. The RNaseH-mediated mechanism of inhibition can occur with phosphodiester or phosphorothioate backbones of the ODN. The upstream cleavage product by RNaseH has a 3′ hydroxyl and the downstream cleavage product has a 5′ phosphate. Information has accumulated that the RNaseH mechanism lacks great specificity with fully cleavable ODNs in that only a small number (≤5 nt) of annealing nucleotides (nt) are sufficient to support target phosphodiester cleavage [7, 8]. This results in substantial off-target effects and has sponsored the development of second-generation agents that have modified backbone and sugar chemistries. Many of these chemistries act to increase the affinity of the ODN to the target RNA. On the other hand, they do not allow RNaseH-mediated cleavage. In pure form, such agents may not have high efficacy when transduced into mammalian cells, indicating that the physical blockade mechanisms are not the most potent. Second-generation chimeric antisense molecules were then engineered that contained the modified chemistries for the backbones and sugars but also a central core of deoxynucleotides that permit RNaseH cleavage. Such chimeras have increased potency on the basis of catalyzing RNaseH attack on a target and specificity because of the strength of binding to the target [7, 9, 10]. During early development, RNaseH activity appeared to be the dominant mechanism of AS inhibition [11]. More recently, a combination of mechanisms is thought to be embraced depending upon the chemical nature of the ODN [12]. An effective AS PTGS agent requires an accessible region in the target RNA and especially strong binding energy of the ODN to the target RNA. The lifetime of the bound ODN: target state must be sufficiently long to embrace the natural kinetics of RNaseH and its stoichiometry-dependent kinetics or must be sufficiently long to impair translation of substantial numbers of cognate protein molecules. The AS reaction scheme can be simply represented as follows:

(1) $$\text{ODN}+\text{RNA}\overset{k_{1}}{\underset{k_{-1}}{⇄}}\text{ODN}:\text{RNA}.$$

The dissociation constant (*K*_*d*_) is given by

(2) $$K_{d}=\frac{\left[\text{ODN}\right]\left[\text{RNA}\right]}{\left[\text{ODN}:\text{RNA}\right]}=\frac{k_{-1}}{k_{1}}.$$

For AS, Rz, and RNA_i_, on rates (*k*_1_) of interaction between the PTGS agent and the target mRNA are limited by the expected rate of forming a nucleic acid double-stranded helix in solution from two (idealized) random coils (estimated at 5 × 10^7^ M^−1^ min^−1^), with the assumption of preexisting regions of single-stranded accessibility able to support immediate base pairing [13]. However, measured AS annealing rates vary more than dissociation rates and appear responsible for the profound range of *K*_*d*_ values that span several orders of magnitude against a single-target mRNA [14–16]. The fact that the ON rates have such a wide variation is likely an index of the varying landscape of accessibility at different regions in a folded target mRNA or potential inaccessibility in the structure of the AS ligand that limits annealing (e.g., [15, 17–19]). OFF rates (*k*_−1_) are typically many orders of magnitude smaller than on rates such that *K*_*d*_ can be approximated by the simple ratio of rates. The net energetic effects of the AS-binding process reflect the losses of potential inhibitory secondary structures in the target or AS ligand and the gain achieved by the annealing event. The strength of binding or the free energy (Δ*G*) of the AS dissociation reaction is represented as

(3) $$\Delta G=-{RT\ln\;K}_{d}.$$

Δ*G* at a particular temperature can be calculated from nearest neighbor tabulations of Δ*H* and Δ*S* [20, 21]. Δ*G* can then be used to calculate *K*_*d*_ from which *k*_−1_ (dissociation rate) can be calculated. *k*_−1_ can then be used to calculate the lifetime (time constant) of the AS bound state

(4) $$\tau_{-1}=\frac{\ln\;2}{k_{-1}}=\frac{0.693}{k_{-1}}.$$

Assume the lifetime of a cellular mRNA target that codes for a relatively abundant protein is 10 hours. In order to manifest significant target knockdown, an AS agent must remain stably bound to the target mRNA for a period at least as long as the target mRNA lifetime. The lifetime of the target: ODN complex allows RNaseH-mediated cleavage of the target mRNA or translation arrest. For an mRNA with a mean 10 hr lifetime, *k*_−1_ should be on the order of 1.2 × 10^−3^ min^−1^, and *K*_*d*_ would be 23.1 picoMolar. AS-binding affinities can vary over several log orders depending upon the target sequence and are often not as strong as 23 pM [14–16, 22, 23]. The dominant factor in achieving a successful agent is to first identify the regions in the target mRNA that are indeed accessible to annealing (see [18, 19]). The length of the ODN and the backbone chemistry should be chosen appropriately to achieve a sufficiently negative Δ*G*, which can be calculated from nearest neighbor frequencies. Web databases for AS ODN effectiveness studies are available [24–26]. The *in vitro *binding capacity and affinity of AS agents to target mRNAs appears to correlate with knockdown potential in live cells [18, 19]. That local target accessibility is a major limiting variable *in vivo *has been shown by engineering a single AS annealing site into a reporter target mRNA in different local structural contexts and then testing knockdown by a single AS ODN relative to control [27]. There was marked changes in knockdown by the single AS ODN when its target sequence was present in different secondary structural contexts. 

Vitrovene (fomiversen, Isis-2922) (Novartis, ISIS), currently the only FDA approved (August 1998) PTGS agent (antisense) for human use (CMV retinitis), is a 21-mer phosphorothioate AS ODN that anneals to the coding region of the mRNA transcribed from the major immediate-early (IE55) gene of the CMV genome [28–30].

### 1.2. Ribozymes

 General reviews on the ribozyme are available [31–38]. A ribozyme is a catalytic RNA. The chemistry of RNA is sufficiently robust that it can fold into structures that permit specific phosphodiester bond cleavage in other target RNAs. There are several forms of ribozyme that have been identified. We focus on the hammerhead ribozyme (hhRz), because it has the most versatile set of cleavage sites (NUH↓, where N = G, C, U, A; H = C, U, A), because a large knowledge base is established for this RNA enzyme, and because the internal equilibrium of the reaction is strongly biased toward cleavage (*k*_2_) as opposed to religation (*k*_−2_) (>100 : 1). The hairpin ribozyme (hpRz) recognizes a broad set of target motifs, but has a religation rate that exceeds cleavage rate (10 : 1) such that religation is favored over cleavage [39, 40]. These issues complicate its potential for therapeutics, because there are fewer places to cleave a tightly compact target and cleaved target products can be relgated by the same agent unless they are displaced rapidly. For the hhRz there are an average of one NUH↓ cleavage site every twelve nts (1/4 × 1/1 × 1/3 = 1/12). Therefore, even an average size mRNA has a rich abundance of potential NUH↓ cleavage sites. This increases the probability for having a potential cleavage site in a rare region of target accessibility. Different NUH↓ cleavage sites demonstrate variation in the rate of cleavage with the two naturally occurring motifs (GUC↓, GUA↓) having the greatest intrinsic cleavage rates [41–44]. In addition to the NUH↓ cleavage motif hhRzs can be designed to cleave at NHH↓, but the catalytic rates are substantially reduced compared to high level GUC↓ motif [32]. All hhRzs cleave a phosphodiester bond to leave an upstream product terminated at the 3′ end with a cyclic 2′3′ phosphate and a downstream product terminated at the 5′ end with a hydroxyl group. Once the target mRNA is cleaved by the hhRz, the fragments are more readily degraded by exonucleases in the cell because of the loss of the polyadenylation signal at the 3′ end of the upstream fragment and the loss of the cap on the 5′ end of the downstream fragment.

**Figure 2:**
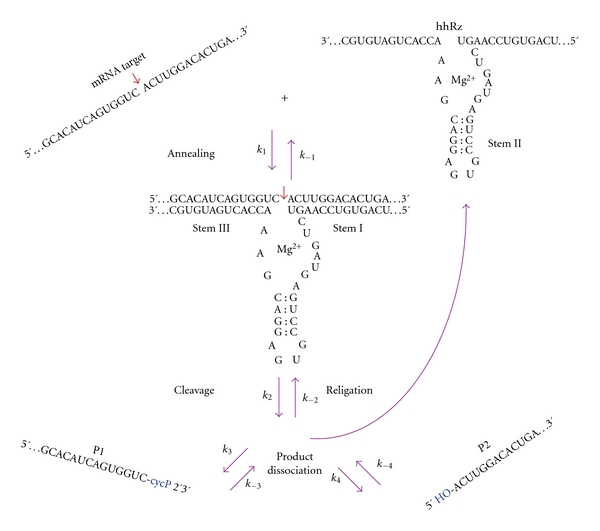
Ribozyme mechanism. A schematic reperesentation is shown with a simplified accessible region in a target mRNA with a cleavable hhRz NUH↓ motif, (GUC↓). The hhRz, drawn in an open enzymatically patent state, binds to the accessible target region by Watson Crick base pairing. Annealing precisely aligns the phosphodiester bond of the H residue (C here) with the enzymatic core of the catalytic RNA. The annealing reaction has an equilibrium specified by the ratio of rates *k*_1_ and *k*_−1_. Chemical cleavage (*k*_2_) occurs to yield two products which remain bound to the AS flanks of the hhRz. Each product leaves with its own characteristic equilibrium determined by the strength of binding to the hhRz AS flanks. Product dissociation permits the enzyme to collide with another substrate and initiate subsequent rounds of catalysis as a Michaelis-Menten enzyme to achieve catalytic target turnover characterized by *k*_cat_/*K*_*m*_.

A simplistic reaction schematic for the hhRz is shown (Figure 2). The hhRz folds into a conformation which is stabilized by Stem II. In its *trans *format, which is used for gene therapeutic purposes, the two antisense flanks form Stems I (5′ AS flank) and III (3′ AS flank) upon annealing to the target RNA. Annealing sets the stage for conformational changes (Rz′) that prepare and align the enzyme core with the phosphodiester bond at the target cleavage site. The H nt of the NUH↓ cleavage motif does not hydrogen bond to the hhRz. Upon cleavage the two products (*P*1, *P*2) must dissociate from the AS arms of the hhRz in order to free the hhRz to anneal to another target RNA and promote true catalytic turnover of substrate:

(5) $$\begin{array}{cc} \text{Rz}+\text{RNA} & \overset{k_{1}}{\underset{k_{-1}}{⇄}}\text{Rz}:\text{RNA}\overset{k_{ES}}{\underset{k_{-ES}}{⇄}}{\text{Rz}}^{\prime }:\text{RNA}\\ & \overset{k_{2}}{\underset{k_{-2}}{⇄}}\text{Rz}:P1\cdot P2\overset{\;}{\underset{\;}{⇄}}\text{Rz}+P1+P2 \end{array}$$

As for AS, the initial dissociation constant (*K*_*d*_) is given by

(6) $$\begin{array}{c} K_{d}=\frac{\left[\text{hhRz}\right]\left[\text{RNA}\right]}{\left[\text{hhRz}\right]:\left[\text{RNA}\right]}=\frac{k_{-1}}{k_{1}} \end{array}.$$

Like AS, Rz and RNA_i_ have ON rates (*k*_1_) that are typically limited by the expected diffusion-limited rate of forming a nucleic acid double-stranded helix in solution from two (idealized) random coils (estimated at 5 × 10^7^ M^−1^min^−1^). Again, association rates are typically orders of magnitude lower than this index. OFF rates (*k*_−1_) are typically many orders of magnitude smaller than ON rates such that *K*_*d*_ can be approximated by the simple ratio of rates. The free energy of the Rz dissociation reaction is represented as in (3) above. Δ*G* at a particular temperature can be calculated from nearest neighbor tabulations of Δ*H* and Δ*S* [20, 21]. Δ*G* can then be used to calculate *K*_*d*_ from which *k*_−1_ (dissociation rate) can be calculated. *k*_−1_ can then be used to calculate the lifetime (time constant) of the Rz bound state as in (4) above. The total AS flank lengths (Stem I + Stem III, H does not hydrogen bond) of the Rz should be no more than 12–16 nts, depending upon the sequence context, in order to achieve a full annealing energy of between −12 to −16 kCal/mole [45]. HhRzs that bind too tightly to target RNA will have slow OFF rates prior to chemical cleavage (rate limiting for the ideal hhRz performance). Slow initial OFF rates could result in a loss of specificity for the intended target because chemical cleavage could occur if an NUH↓ site of an unintended target happened to be centrally placed within the AS flank span. The likelihood that an unintended target could precisely position itself on a given hhRz for cleavage at an NUH↓ site is, in fact, low, unless the unintended target had almost precise sequence identity to the intended target. This factor has been presented as a factor for hhRz specificity [46]. In addition, hhRz catalytic function is intolerant to base-pair mismatches near the core of the enzyme [47], which would act to decrease cleavage of bound nontarget mRNAs that do not have precise sequence specificity for annealing; in fact, this attribute of the hhRz can be used as a component of a therapeutic strategy to suppress mutated versus normal target mRNAs in hereditary diseases (see below). Off-target effects with a hhRz would more likely result from pure AS effects independent of catalytic chemical cleavage. With an optimum total antisense flank length for catalysis on the order of 12–16 nt, the stable annealing of unintended targets with mismatches relative to the hhRz is expected to occur with low probability. The expected specificity of the hhRz is a considerable distinction from the mismatch tolerant AS or RNA_i_ processes. 

Another challenge with the hhRz is the issue of product inhibition. If the two products cannot melt off of the antisense flanks of the hhRz after cleavage at physiological temperature, or one product is delayed in leaving, then the hhRz will be trapped in association with cleaved target and unable to recognize and anneal to subsequent target RNA molecules. This problem impacts catalytic turnover or enzyme efficiency (*k*_cat_/*K*_*m*_). A kinetic model exists for the hhRz that can greatly assist in the design of antisense flanks that permit energies of annealing sufficient to allow the hhRz to bind long enough to permit chemical cleavage (*≅*1/min) but not too long to promote product inhibition [13]. It is important to determine the extent to which target knockdown by a hhRz is due to catalytic, antisense, or catalytic antisense effects. There are several mutations that can be made at key residues in the enzymatic core of the hhRz, which are known to completely obviate catalysis (e.g., G5C, G8C, G12C [41, 44, 48]). Comparing the level of target knock down (RNA or protein or both) by a fully catalytic hhRz compared to a mutated hhRz should allow sufficient information to determine the extent to which the hhRz is performing catalytically, which is the desired outcome. A hhRz with true catalytic performance *in vivo* can knockdown significantly more target molecules in a given epoch of time than a hhRz that does not have this capacity (e.g., pure AS effect without cleavage or a catalytic antisense effect with annealing and cleavage but no product release and turnover). Hence, hhRzs that demonstrate catalytic turnover in live human cells require lower expression levels to achieve the same levels of target knockdown than those that do not have Michaelis-Menten turnover potential. Lower levels of PTGS agent expression are expected to decrease the potential for cellular toxicity and off target effects. 

A relatively stable mRNA is a good target for gene silencing, because hhRzs are relatively slow enzymes. The intrinsic cleavage rate is maximal against small unstructured substrate RNAs and on the order of 1/min, which is several orders of magnitude slower than proteinaceous enzymes. Structured targets typically have slower cleavage rates. Because of the slow speed of catalytic RNAs, the intrinsic degradation kinetics of the target RNA (without the hhRz) and with the hhRz RNA must be considered. It is important to consider the lifetime of the target mRNA in its dominant locale within the cell. Targets that have short lifetimes (e.g., pulse transcribed mRNA with rapid turnover such a cell-cycle control genes) may be difficult to attack with current hhRzs, because the targets intrinsically degrade at a rate that cannot be practically impacted by a hhRz. One will want to choose targets carefully to insure that there is sufficient time for enzymatic turnover within the cell at expression levels of the PTGS agent that are not toxic. We would recommend target mRNAs that have lifetimes on the order of several hours. Fortunately, most autosomal dominant disease genes and normal genes transcribe fairly stable mRNAs as potentially validated targets for PTGS therapeutics. These typically code for signaling, structural, or enzymatic proteins in photoreceptors and RPE cells. Any Rz acts kinetically by providing an additional component to the intrinsic degradation rate for a target RNA. The total rate of degradation of the target mRNA is the sum of the intrinsic and Rz-induced degradation rates (*k*_cat_ = *k*_int _ + *k*_PTGS_). Clearly, if the intrinsic degradation rate is much faster than the rate of intracellular Rz catalysis, then *k*_cat_≅*k*_int _, and there can be no significant knockdown of target RNA and protein mediated by the PTGS agent. An hhRz or an RNAi can be most effective if *k*_PTGS_ ≫ *k*_int _. On face value, this substantially restricts the types of mRNAs that can be suitable targets. RNAs with very short half-lives, such as those coding for transiently induced transcription factors, are unlikely to be viable targets because *k*_PTGS_ ≈ *k*_int _ or *k*_PTGS_ < *k*_int _. Therefore, before embarking on the development of a PTGS agent for a particular target, it is prudent to have knowledge regarding the intrinsic degradation half life of the target mRNA in the cells in which gene therapy would need to be administered.

A ribozyme designed to cleave the mRNA for proliferating cell nuclear antigen [49] was recently tested in a Phase I clinical trial for proliferative vitreoretinopathy [50].

### 1.3. RNA_i_ Technology

**Figure 3:**
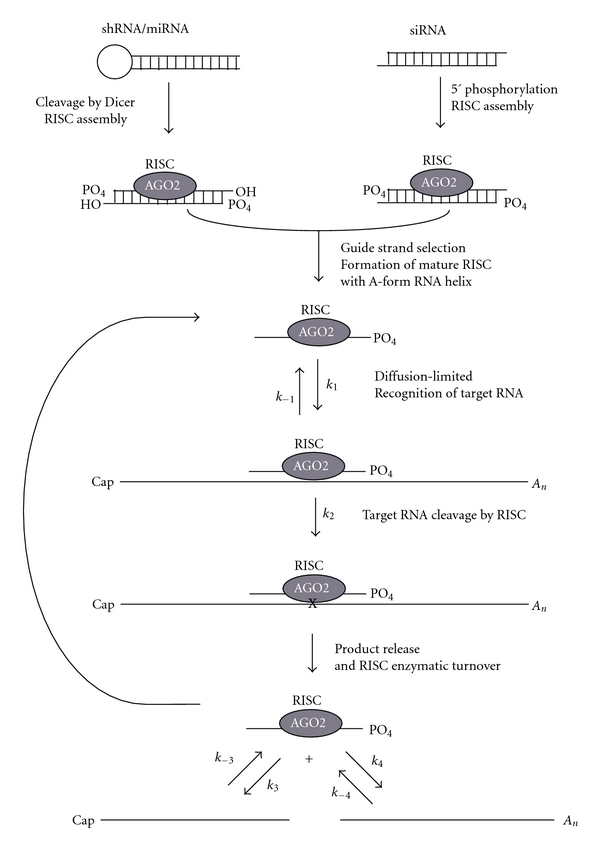
RNAi mechanism. An expressed RNA hairpin (shRNA) is cleaved first by Dicer III to a double-stranded RNA of 21 nt with 5′ phosphorylated ends. A pri-miRNA is processed in the nucleus into a pre-miRNA by Drosha, leaves the nucleus, and is further processed by Dicer in the cytoplasm or as part of RISC. Or a transfected or transduced siRNA is phosphorylated at each 5′ end. The short dsRNAs are incorporated into the RISC complex, and the antisense strand (guide strand) is selected on the basis of engineering weaker 5′ energy than 3′ energy. The passenger strand is displaced. The guide strand is organized into RISC as an A-form *α*-helix within Ago2, which is the RNA endonuclease of RISC. By diffusion limitations, loaded RISC searches for a complimentary partner to its antisense element in the transcriptosome. Upon collision, kissing complex formation and full annealing, the target RNA is positioned for endonuclease cleavage by Ago2. After cleavage, it is thought that ATP hydrolysis occurs, which provides helicase energy to strip the products from the Ago2 cavity in order to prevent product inhibition on RNAi. Product release then frees the charged RISC to seek other target mRNAs for subsequent rounds of Michaelis-Menten turnover.

Recent reviews will serve to orient the unfamiliar reader [51–53]. RNAi refers to an evolutionarily conserved phenomenon where double-stranded RNA (dsRNA) mediates the sequence-specific cleavage of target RNA using cellular machinery (Figure 3). In mammalian cells, RNAi is triggered by 21–23 nt RNA duplexes with symmetric 2 nt 3′ overhangs and 5′-phosphate termini called small interfering RNA (siRNA) [54–56]. These siRNA duplexes are processed from longer dsRNA by the ribonuclease III enzyme Dicer [57]. Dicer processed siRNA duplexes associate with a multiprotein complex known as the RNA-inducing silencing complex (RISC), and one strand of the duplex is loaded into RISC to serve as the AS guide strand. Within RISC, the guide RNA strand is bound by the Argonaute 2 protein that contains an amino-terminal Piwi Argonaute Zwille (PAZ) domain and a carboxy-terminal PIWI domain containing the catalytic RNA slicer site [58]. The PAZ domain recognizes and anchors the 3′ overhang of the duplex [59–61] while the PIWI domain anchors the 5′ end of the guide RNA [62]. The guide strand then adopts a A-form helix that extends along a channel in the PIWI domain, aligning the scissile phosphate of the target strand with the slicer catalytic site one helical turn away from the 5′ anchored end [63]. The PIWI domain is similar in structure to RNaseH. After RISC cleavage, the upstream product has a 3′ hydroxyl and the downstream product has a 5′ phosphate. 

Despite the association of a cellular protein complex, effective gene silencing is still not realized with many siRNA or expressed short hairpin (shRNA) sequences [64]. Three crucial kinetic parameters are strongly implicated in the ability of a given siRNA sequence to effectively promote gene silencing in physiological conditions: the loading of the correct antisense RNA guide strand into RISC, target mRNA site annealing, and RISC reloading. These parameters are affected by sequence-specific problems. For the first parameter, loading of RISC, the thermodynamic stability of the RNA ends has been shown to be the major determinant of which strand of the siRNA duplex is incorporated into RISC. Theoretically, either strand of the siRNA duplex can be incorporated into RISC, but only one strand will be AS for a given sense mRNA target. The discovery that the strand with the greater thermodynamic instability in the 5′ end is preferentially loaded into RISC has improved the design of successful siRNAs or shRNAs [65, 66] by allowing the preferential loading of the correct antisense guide strand. While the loading of RNA guide strands into RISC is an RNAi-specific problem, the problem of the limits of target mRNA site accessibility and annealing that occurs for AS and ribozyme PTGS agents also affects efficacy of siRNA sequences [67–74]. Fundamentally, the Watson-crick base pairing that is required for all of these technologies profoundly limits the number of target mRNA regions that will support effective gene silencing. Like AS and ribozymes, target recognition for RNAi also seems to proceed by diffusion [71], with the guide strand of the RISC complex encountering sites nonspecifically until proper annealing with the target site forms the necessary geometry for RISC cleavage. Target recognition is dominated by the 5′ region of siRNA, which nucleates binding of target RNA with RISC and contributes to the overall strength of binding between the target RNA and RISC. The 5′ region of the siRNA (2–8 nt) has been called the “seed” sequence. The annealing of the central and 3′ regions are important for establishing the A-form helical geometry that is needed for efficient central cleavage [75, 76]. Although RISC proceeds with greatest activity when it anneals to a fully complementary target, it can still cleave RNA targets with mismatched bases, especially in the 3′ end. Even with such mismatches, the RNAi mechanism can also promote translational inhibition. The toleration of mismatches gives rise to the significant off-target effects of potential RNAi therapeutic agents (see below). In *D. melanogaster* embryo lysates, target annealing and cleavage by RISC are both ATP-independent steps. It is only the release of the target after cleavage that requires ATP [75]. The expected increased catalytic efficacy of RNAi compared to ribozymes is most likely due to the increased OFF rates of products that is facilitated by RISC. This step may be slower in humans as the Drosophila RISC enzyme seems to have a higher catalytic efficiency despite similar *K*_*m*_ values [77]. Recent studies indicate that the RISC complex can be saturationally inhibited by other competing siRNAs and that the loading (1 hr) and clearance (12 hrs) of the RISC complex have distinct kinetic rates [78]. While the suggestion that RNAi is more potent than AS or Rz modalities, RNAi still shares with all PTGS modalities the same major problem of the intrinsic limits of target inaccessibility, with the initial challenge being to identify rare accessible regions. Few studies have compared RNAi potency to other modalities at sites in target mRNAs that are predetermined to be accessible or inaccessible *in vivo*. Even if intrinsic potency is greater for RNA_i_, the potential for off-site and toxic effects of RNA_i_ may make ribozyme or perhaps AS better choices for therapeutic PTGS development. 

Recent discoveries continue to reveal the complexity of the machinery involved in the RNAi mechanism, and great care must be taken to evaluate potential RNAi therapeutic agents. For RNA therapeutics to be safe clinically, they must have specificity. There are an increasing number of reports about off-target knockdown effects by RNAi [79–83], some of which have induced toxic effects [84]. This likely results because of the tolerance of RISC to mismatches in bound target RNAs and a decrease in specificity for intended targets. siRNA activation of interferon response genes has occurred [85] as well as activation of the immune system [86]. Recent serious concerns over RNAi safety were raised due to death of mice secondary to RNAi saturation of a nuclear exit pathway (exportin-5) used by micro-RNA [87–89]. Therapeutic interference with natural and essential functions of micro-RNAs, such as differentiation, cell-cycle control, and gene expression, could also cause serious deleterious consequences. These findings raise serious concern about potential toxicity of RNAi in human clinical trials. In addition to off-target knockdown concerns, a recent study also revealed the potential for sequence-independent knockdown of an RNAi target unrelated to off-target immune effects. Anti-angiogenic siRNAs were targeted to VEGF or its receptor for the treatment of choroidal neovascularization (CNV) in age-related macular degeneration. The siRNAs showed a suppression of CNV that was caused by a class effect of 21-nucleotide double-stranded RNA sequences stimulating cell-surface Toll-like receptor 3 that lead to an induction of interferon-gamma and interleukin-12 rather than a specific knockdown of VEGF or its receptor [90]. A siRNA database is available for the interested reader [91]. 

Micro-RNAs (miRNAs) are noncoding regulatory RNAs expressed in mammalian cells generally from RNA pol-II promoters as primary miRNAs (pri-miRNAs). Pri-miRNAs are processed in the nucleus by the endonuclease Drosha to form pre-miRNAs, which are derivative hairpin RNAs that are transported by Exportin-5 to the cytoplasm. There they are further processed by Dicer into 21–23 bp dsRNAs that enter the RISC processing pathway. miRNAs control development, gene expression, cellular differentiation, growth regulation, and many have been identified in human cells [92]. miRNAs appear to be the native substrates of the evolutionarily conserved RISC RNA_i_ pathway. miRNAs, like other RNA_i_ modalities, can promote cleavage of target mRNAs if there is full binding to the target mRNA, or translational inhibition when bound by seed sequences, but with mismatches, to 3′ UT sequences. Recent efforts have sought to create designer miRNAs in which a particular native human miRNA, which is expected to have its own intrinsic set of target mRNAs, is engineered to create potential for annealing to a disease target mRNA. Early data suggest that this approach may yield both potency and decreased potential for toxicity, because lower levels of expression of the miRNA are achieved [93, 94]. However, the design and embedding of PTGS agents as chimeras within usurped native human miRNAs that naturally interface through RISC to modulate critical cellular functions may create risk. More studies are needed to establish both effectiveness and safety of this approach. Clearly, miRNA evolution achieved specific RNA structures that were processed and reduced to functional siRNAs inside cells. The insertion of an alternative nonnative targeting sequence into a larger miRNA embraces substantial new biophysical constraints. How can one insure that the targeting siRNA is properly spliced from a larger RNA when multiple conformational states of the miRNA chimera can exist and these might affect how Drosha and Dicer process the expected target sequences?

## 2. Variables and Challenges in Therapeutic PTGS Development

### 2.1. Overview

A PTGS agent is designed to suppress the translation of a particular target mRNA into its cognate protein. This may occur through tight annealing of the PTGS agent to the target mRNA which stalls translation at the ribosome. Or, it may occur through annealing and cleavage of the target mRNA which promotes more rapid degradation of the target mRNA, to decrease the steady state concentration of the target mRNA and suppress translation at the ribosome and hence the steady state level of the cognate target protein. In the context of a therapeutic PTGS, the particular mRNA/protein targets must be validated for a given disease state, such as a retinal degenerative disease. Validation means that the expression of the specific target mRNA/protein has been strongly associated with the emergence of a particular disease state. For example, in an autosomal dominant form of hereditary retinal degeneration such as retinitis pigmentosa, the expression dose of mRNA from the mutated allele may generate a protein which has toxic gain of function for the cells in which it is expressed. This toxicity may promote stress and ultimately apoptosis. At least, early in the disease process, it is rational to select the mutated mRNA as the validated target for therapy of such a genetic disease. If the mutant mRNA and toxic protein can be reduced, this outcome is expected to ameliorate cellular stresses and reduce the probability of apoptosis and the coincident loss of cellular and visual function. Similarly, in certain retinal degenerative conditions, such as age-related macular degeneration, rational therapeutic PTGS strategies could potentially involve the reduction of levels of wild-type gene expression. 

PTGS agents operate biophysically within the functional context of cellular housekeeping functions to reduce levels of specific target mRNAs and proteins. The critical *variables* in the design of efficacious potentially therapeutic PTGS agents are not specific to retinal or other ocular diseases and in fact, have largely emerged from research not specific to ocular disease states. Therefore, we have attempted here to represent to the reader the biocomplexity of these challenges garnered from the PTGS literature at large, because the rules identified are equally relevant and essential for development of such PTGS agents for human retinal or eye diseases. Hence, we have specifically not attempted here to review the emerging PTGS literature for retinal or ocular degenerations. Our focus here is on the variables that influence development and efficacy of a PTGS agent itself (the drug or R_x_) rather than on the means of delivery of such an agent to the affected cells (e.g., through a vector or chemical design). When discussing the core *strategies *for therapeutic PTGS development, which did strongly emerge from early studies applying such agents to hereditary retinal degenerations, we touch on studies that lead to these strategies. 

Successful design of a PTGS agent, be it AS, Rz, or RNAi, involves biocomplexity at the biophysical, biochemical, and cell biological levels. A target mRNA molecule is folded into dense secondary and tertiary structure, it is coated with heterogeneous proteins, it undergoes dynamic conformational fluctuations, and it resides in unique intracellular compartments with different lifetimes (nucleus, cytoplasm, ribosomes, etc.). These target mRNA factors *severely* constrain the locations in the RNA target that are accessible to the annealing of a colliding small PTGS agent and the range of timescales and spatial environments available for small PTGS ligand attack. In addition, the PTGS must be able to achieve a ground state conformation in which it is fully available to interact with and anneal to exposed regions of the target mRNA (molecular recognition). For Rz PTGS agents, the catalytic RNA bound to the target RNA must be able to undergo conformational transitions that promote RNA chemistry-based target cleavage. The structure-function properties of Rz-based PTGS agents become especially difficult when the Rz is embedded in a larger chimeric RNA to provide cell trafficking, stability, and high levels of expression. PTGS biocomplexity is a multivariate problem that is a major factor in the slow entry of nucleic acid knockdown agents into the pharmaceutical market despite obvious clinical potential. RNA structural biology greatly limits PTGS therapeutic strategies. In this paper we present the variables that must be understood for successful development of a PTGS agent. We present aspects of target RNA biology that will convince the reader about the biocomplexity of PTGS development. We present the different strategies and approaches of how PTGS agents can be used therapeutically for hereditary and degenerative diseases of the retina or eye and the relevant variables in the design of materials for such strategies. While RNA-directed drugs are still largely on the horizon, we briefly describe some high throughput screening (HTS) approaches that are expected to greatly influence further development of PTGS agents. Recent emergence of tools to address difficult scientific issues underlying the biocomplexity of the transcriptosome and RNA structure/function offer substantial hope that the dawn of clinical translation of RNA-directed drugs is visible in the near future. Use of HTS approaches to relieve bottlenecks in PTGS development is dealt with in detail in a separate review [95].

### 2.2. Common Variables That Affect Efficacy of PTGS Agents

There are five critical variables that are essential to understand in order to design PTGS agents that are efficacious *in vivo*. Limitation of any single property is sufficient to completely obviate functionality of the PTGS agent. *First*, the PTGS agent and its target RNA must be in the same cellular locale or compartment in order to allow for potential annealing interaction. *Second*, the PTGS agent must be in sufficient concentration to drive an adequate second-order collision frequency which is essential to secure annealing. *Third*, the target RNA must present an accessible and kinetically stable single-stranded platform at physiological temperature in order for the PTGS agent to anneal. *Fourth*, the PTGS agent itself must be in a conformational state that permits direct and full annealing to the target RNA. * Fifth*, for Rzs and RNAi, the cleavage products must dissociate rapidly from the enzyme to insure potential for enzymatic turnover (Michaelis-Menten kinetics). 

#### 2.2.1. Colocalization

 In all cases, the PTGS ligand and the target RNA must colocalize in precisely the same spatial environment within the living cell, and on the same timescales, to support frequent collisional interactions that may result in kissing complex formation and full annealing [96–98]. RNAs (both target and PTGS agent) move along trafficking streams inside the human cell and have lifetimes at each stopping point along the way to their final destination(s). Hence, expressed RNAs may distribute among different spatial locales within the cell. Most mRNA targets for PTGS will spend the largest amount of their intrinsic lifetime in the cytoplasm, where they are diffusing, translated on the ribosome, or stored in RNA granules. While it is easy to appreciate that a PTGS agent that traffics to the nucleus will be unable to effect knockdown of a cytoplasmic mRNA target, more subtle issues are that both target mRNA and PTGS RNA agent could be in the same macroscopic compartment (e.g., cytoplasm) and yet not colocalize, because they do not occupy the same cellular RNA zip code, or that the lifetimes of the target and PTGS agent in a given locale are so disparate that meaningful second-order collision frequency is not probable. There are both gross macrocompartments and microcompartments within those in which mRNA targets and PTGS agents will need to colocalize for effective interactions. RNA zip codes are known to exist, and play a role in cellular RNA trafficking, storage, or to sponsor RNA: RNA interactions [99, 100]. The ideal situation for gene therapy is if the PTGS agent is engineered to occupy the same specific RNA zip code within cells as its cognate target, and that the lifetime or stability of the PTGS agent in the cell within the preferred locale is on the same order or greater than the lifetime of the target mRNA in the same spatial locale. In order to achieve colocalization with the target mRNA it may be beneficial to embed the PTGS agent into a carrier RNA to create a chimera. The carrier RNA (e.g., tRNA and VAI RNA) has established structure and function, is expressed to high levels in the cell, and has known trafficking properties that lend itself useful for colocalization of the PTGS agent with its target. Embedding a PTGS agent within a carrier RNA certainly adds to complexity of structure/function of the PTGS agent, which is an area that has not yet received much investigative effort.

#### 2.2.2. PTGS Concentration and Diffusion Limitations

 The PTGS agent must be present at sufficient concentrations in the same cellular locale as the target RNA to allow a fast and effective diffusion-limited second-order ON reaction rate (*k*_1_) with the target RNA. While we normally think of enzyme reactions in macroscopic terms, with the substrate in substantial excess (Michaelis-Menten condition), it is prudent to consider the actual concentration of a target RNA inside a cell. Target RNAs are typically expressed in low numbers inside the cell. Even from a relatively strong promoter (e.g., human rod opsin) an estimated steady-state level of approximately 2500 mRNA molecules resides in a cytoplasm of total volume 1.75 picoliters (simplifying assumption of a spherical cell with 15 *μ*m cell diameter and spherical nuclear diameter of 3 *μ*m and with no excluded cytoplasmic volumes) would yield a steady-state concentration of 2.4 nM. With weaker promoters, 250 mRNA molecules would yield a steady-state concentration of 240 pM, and 25 mRNA molecules could yield a concentration of only 24 pM. Even when the target mRNA is relatively abundant, these estimates indicate low cellular concentrations for the substrates (targets) of an initial PTGS annealing reaction. In addition to the target mRNA being in low concentration, it is also expected to be large with slow cellular diffusional coefficients [101]. Hence, the PTGS agent must be expressed or delivered in sufficient concentrations in the correct cellular compartment to promote an efficient collision rate with the target mRNA in order to promote a rapid second-order annealing reaction [102, 103]. The time scale needed for the functionality of the PTGS must also be embraced. For example, a rapidly degraded and intrinsically short lived mRNA that codes for a short lived protein involved in cell cycle regulation (e.g., a transcription factor), may have a half-life on the order of minutes and be expressed in low concentrations. It would be difficult for any PTGS technology to modulate the knockdown of such an mRNA and protein simply, because the kinetic action of the agent (e.g., Rz and RNAi) may be too slow to modulate an intrinsic process of mRNA degradation that is already rapid (*k*_cat_ = *k*_int _ + *k*_PTGS_≅*k*_int _).

#### 2.2.3. Target Accessibility

Regardless of the experimental approach, large bonafide regions of stable accessibility in target RNAs are rare in RNAs of any substantial size. The biocomplexity of the RNA target is the prime and profound limiting variable in the successful design of PTGS agents. It is the factor that limits successful PTGS design of any type (AS, ribozyme, RNAi) [104]. The secondary and tertiary structures of the folded mRNA impose a severe limitation to identifying suitable accessible regions for PTGS attack. 

**Figure 4:**
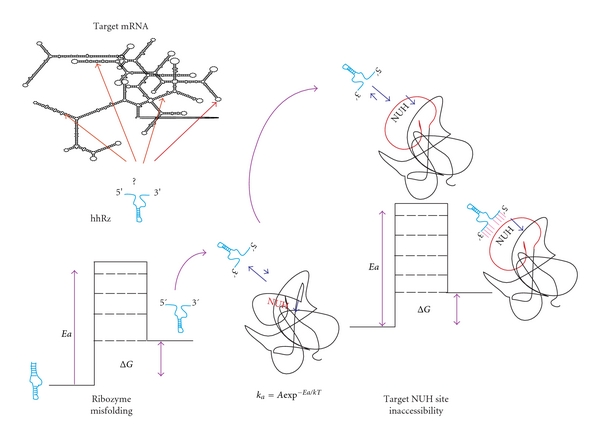
Secondary and tertiary structure as a limiting variable in PTGS efficacy. Energy diagrams are presented for both the target mRNA and the PTGS agent. The folded target mRNA has a site targeted for annealing which is buried in secondary or tertiary structure. The rate of unfolding of this region is determined by the activation energy required for conformational transition that leads to accessibility of the annealing platform. For the folded target RNA to be accessible it must present a single-stranded annealing platform(s) at its surface to allow annealing with the PTGS agent upon intermolecular collision. Buried regions of the RNA that are targeted must wait at physiological temperatures for relaxation of secondary and tertiary structure in order to present an annealing platform. Many regions are expected to never be exposed. The Arrhenius rate provides an estimate of how long it takes for a single-stranded platform to emerge at physiological temperature (310°K = 37°C) and is dependent upon the activation energy (*Ea*) of the transition. Likewise, any internal secondary structure of the PTGS agent itself can prevent annealing to target or slow catalysis and impact efficacy. Melting of inhibitory secondary or tertiary structure in the PTGS agent then can allow exposure of the antisense flanks to support annealing to the target mRNA.

For any PTGS agent to be successful it must be able to collide with and anneal to accessible regions of the target mRNA. All successful PTGS technologies require bonafide regions of accessibility in the target RNA at 37°C for human therapeutics. Regions that are inaccessible, due to overriding RNA secondary and/or tertiary structure, or protein binding, will not permit rapid annealing, thus leading to delays in hybridization during the waiting time for local melting of secondary and tertiary structures at physiological temperature, if such melting is thermodynamically feasible (Figure 4). Any delays will decrease the overall observed catalytic rate (*k*_cat_), and thereby reduce the amount of target mRNA that is cleaved within a given time interval such that target protein knockdown is limited. The capacity of the PTGS to anneal is not only dependent upon a kinetically stable accessible single-stranded platform, but also proper orientation for annealing of the accessible platform in the target mRNA to the approaching PTGS ligand in collisional reactions at physiological temperatures. The biophysical nature of the second-order annealing reaction between ligand and target RNA is critical to success but often unconsidered. 

The biocomplexity of RNA target structure is the primary and dominant variable in the success of a therapeutic PTGS agent. Large, accessible, and kinetically stable sites are rare and expected to follow Poisson distribution statistics. Therefore, initial efforts in any PTGS study should embrace the challenge to find the most accessible sites in the target mRNA. In any average size mRNA target, there are too many potential sites to try and attack with any PTGS technology and orders of magnitude insufficient resources to test them all. It is essential to be highly selective if one wants to achieve a PTGS agent that can be brought into preclinical trials in animal models. The first question is how to successfully identify accessibility in a target mRNA? We will discuss several possible means including emerging technologies. It is prudent to represent the complexity of the target RNA. For the sake of demonstration, we will consider two human mRNA disease targets for candidate therapies for autosomal dominant diseases. Human rod opsin (*RHO*) and human bestrophin (*BEST-1*) mRNAs are suitable examples. Both are the subject of over a hundred mutations that cause human retinal degenerative diseases. Most mutations in the *RHO *gene cause autosomal dominant or autosomal recessive retinitis pigmentosa and less commonly autosomal dominant congenital stationary night blindness or retinitis punctata albescens [105, 106]. Most mutations in the *BEST-1 *gene cause juvenile autosomal dominant vitelliform macular dystrophy (Best disease) and less commonly autosomal dominant adult vitelliform macular dystrophy or dominant bull's eye maculopathy [107–109]. The size of the dominant polyadenylated transcripts in the retina are 1.8 and 2.2 kB, respectively, [110, 111]. These mRNAs targets are of average size. The *RHO *gene is expressed exclusively in human rod photoreceptors in the retina that provide dim light (scotopic) vision, and the *BEST-1* gene is expressed exclusively in the retinal pigment epithelium. 

**Figure 5:**
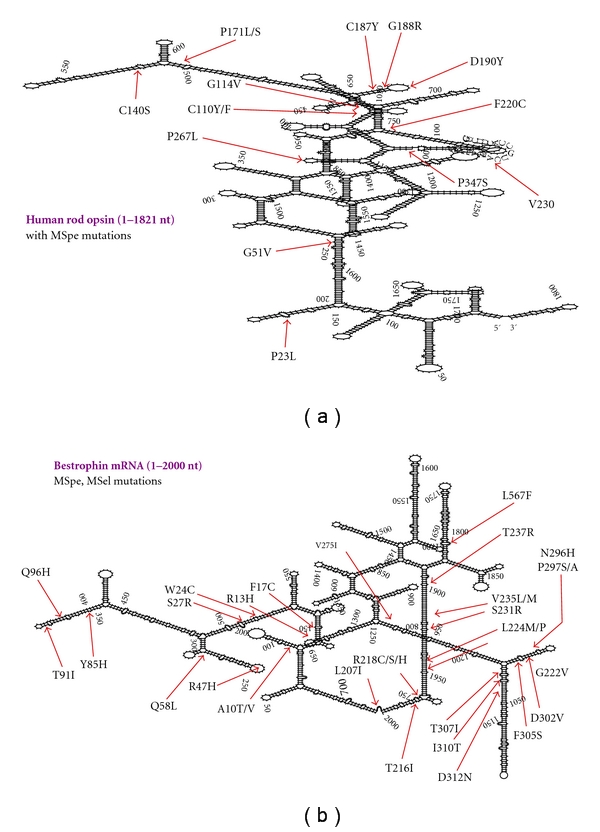
Predicted minimal free energy folding structures of human rod opsin and Bestrophin-1 mRNAs. GeneBank accession numbers for human rod opsin mRNA (NM000539.2) and Bestrophin-1 mRNA (NM004183) are indicated. (a) Human rod opsin mRNA (1–1820 nt) was folded *in silico *with RNA-Fold. The minimal free energy structure is shown. Note the dense secondary structure with only rare single-stranded annealing platforms of any substantial size. Also shown are the locations of human missense mutations that cause autosomal dominant retinitis pigmentosa and that generate *new *hhRz cleavage sites. These are mostly buried in secondary structure, where they would be poor targets for a mutation-specific (MSpe) approach to gene therapy as the PTGS agent would have limited capacity to anneal and cleave only the mutant target mRNA. (b) Human bestrophin-1 mRNA (1–2000 nt) was folded with RNA-fold and the MFE structure is shown. Again, secondary structure is dense with rare single-stranded annealing platforms larger than 10 nt. The locations of human mutations that cause autosomal dominant best macular dystrophy and that generate *new *hhRz cleavage sites for a mutation-specific strategy are shown (MSpe). Most are buried in dense secondary structure, where they would be expected to be inaccessible to annealing of a PTGS agent. Also shown are some mutations located in regions of WT cleavage sites, where they would permit a mutation-selective (MSel) approach to PTGS gene therapy.

There are several computational and experimental approaches that can be applied to the determination of accessible sites in RNA targets (Table 2). Computational approaches are based upon algorithms that predict structures or energy, such as MFold, SFold, and OligoWalk [112]. The interested reader can explore the references associated with the different methods detailed in Table 2. Computational algorithms are available to predict RNA secondary structure. Algorithms such as MFold [113] or the older version RNAFold can be used to obtain images of the secondary structure of a target mRNA. These specific algorithms search for the most stable structure with the minimal (most negative) free energy (MFE). MFE secondary structures of human *RHO *and *BEST-1* mRNAs are shown (Figure 5). Both mRNAs demonstrate densely folded MFE secondary structures. There are rare single-stranded loops of significant size (e.g., >10 nt). This is a common appearance for all RNAs that we have folded computationally. There is always dense secondary structure present, and at best only rare regions containing large single-stranded platforms are present that appear suitable for rapid annealing. We have encountered RNAs that are even more densely folded than those presented here. These represent single-structure snapshots of the folding states of the mRNAs. There are many other potential structures and these MFE structures may not be the native structures in the cell. Nevertheless, even from this simple computational presentation target mRNA accessibility is clearly a dominant limiting variable in design of efficacious PTGS agents. Experimental approaches assessing accessibility support this perspective as well.

Cleavage with intended target site specificity requires that AS, hhRzs or RNAi be directed to accessible single-stranded regions where rapid annealing and generation of the enzyme: substrate complex equilibrium can occur [46, 75, 114–116]. Rapid binding/displacement equilibrium with a significant *k*_OFF_ rate allows mispaired mRNAs to be released before cleavage, which generates specificity for intended targets. Stabilized annealing complexes occurring at off-target mRNAs will decrease specificity. One wonders if the specificity problems reported for AS [117] and the promiscuous specificity of RNAi modalities somehow relates to cellular protein-based stabilization (e.g., through RNaseH or RISC) of mismatched target-PTGS ligand complexes. 

The second-order annealing reaction appears to be the rate limiting step for PTGS agents in the living cell for long lived target mRNAs, and typically requires ten- to several hundred-fold molar excess of ligand over substrate mRNA to achieve good gene suppression [45, 96, 97, 114, 118]. This factor is likely due to the cellular reaction occurring under diffusion-limited conditions. While PTGS agent delivery, expression load, and colocalization with target RNA will affect collision encounter frequency, the major *initial* barrier to intracellular PTGS action is higher order target RNA structure that globally restricts the number of access sites [22, 119–125]. The secondary and tertiary structure obstacle is expected to pose the most serious limitation to the development of PTGS treatment strategies for diverse genetic or non-genetic diseases (see [126, 127]).

#### 2.2.4. PTGS Conformation

 The PTGS agent must itself be in a conformational state(s) supportive of the second order annealing reaction in order to achieve successful target knockdown [37]. Any abnormal intramolecular structure of the PTGS agent itself will create potentially unrecoverable annealing delays (Arrhenius rate of activation) and cause loss of efficacy for a constant ratio of PTGS agent to target mRNA (Figure 4). Regions of self-complementarity within a single-stranded AS ODN or hhRz RNA can occlude the antisense flanks from being freely available to interact with the target mRNA. For a hhRz, it is also possible that the AS flanks intrude into the catalytic domain or into a structured domain (e.g., Stem II). Any perturbation of structure is expected to be potentially deleterious to both annealing and catalytic function. Waiting for such secondary structures to open at physiological temperatures implies an additional rate of reaction that acts to effectively slow the association rate with target mRNA. Since the minimal hhRz has only 4 bp of double stranded secondary structure expected for the entire active enzyme, it is not surprising that alternative conformational states of the hhRz can have marked impact on catalytic efficiency. Attempts to stabilize the hhRz into a proper secondary structure by extending Stem II or adding a stabilizing loop to cap this stem have not lead to improved function [128, 129], perhaps because extension of Stem II has negative impact on the catalytic cleavage rate of the enzyme. Proper structure as well as flexibility may be important for function. For the hhRz, the structure/function problem becomes more challenging with the recent identification of 5′ tertiary accessory elements that form pseudoknots with the Stem II loop and enhance the probability of achieving an enzymatically active state [130–135]. These considerations are focused on the ribozyme sequence itself. If the ribozyme is embedded in a chimeric RNA for strong expression, appropriate cellular trafficking for colocalization with target, and overall stability and lifetime in the cell, the potential for misfolded structures becomes much greater and requires careful rational design for the placement of the PTGS agent within the chimeric RNA (e.g., [136]).

#### 2.2.5. Product Leaving

 AS requires long hold times (high affinities) and hence must be highly stable once hybridized. For AS, a highly stable ES complex must form in order to attract RNaseH for cleavage, and it is necessary to identify stable, accessible regions for PTGS within this cellular milieu. Rzs must have sufficient holding time to permit cleavage but cannot bind too tightly or both loss of specificity for the intended target and product inhibition will occur. Upon cleavage, the upstream and downstream target products must be cleared rapidly from the AS flanks if the hhRz is to have substantial enzymatic turnover of additional target mRNA molecules. Similarly, RNAi requires sufficient hold time for RISC-mediated cleavage and must clear products to promote efficient multiturnover catalysis. For both hhRz and RNAi, too long a hold could be deleterious to specificity and contribute to off-target effects. RNAi is much more susceptible to off-target effects, because the RISC complex is highly tolerant of mismatches with target [75]. RISC in mammalian cells appears to use an ATP-dependent helicase to aid in stripping products, and this gives RNAi an advantage over native ribozymes which depend upon thermal solution properties (*kT*) for product release. In addition, the RISC complex is strongly dependent upon a relatively short “seed” sequence (6-7 nt) within the 5′ end of the guide RNA, which would be expected to interact with a larger set of targets when compared to the entire length of the siRNA (e.g., 19–23 nt). A Rz or RISC that cannot dissociate from the cleaved products of reaction becomes a catalytic antisense reagent without capacity for enzymatic turnover. Product leaving rates must be robust in order to clear the annealing or loading sites to promote next-target annealing. Product leaving rates can be predicted based upon nearest neighbor energetic analysis, which is prudent in early PTGS design (e.g., [13]).

### 2.3. Summary

 The above variables lead to strong directives in approaches to PTGS design. The steady state level of a target mRNA is experimentally invariant as a defined characteristic of a particular target cell. The *only* way to influence the effective statistical collision frequency of the PTGS agent, whether in a deterministic or stochastic process, is to increase the numbers of PTGS ligands *immediately *within the local environment of the target and to keep those ligands relatively small such that they have substantial diffusional rate relative to the larger target RNA (expected to have a slower diffusional rate in the cell). Smaller size of the PTGS agents can also facilitate probing of targets in which the annealing site is found in recessed surface features of the tertiary structure. Successful knockdown of target requires both strong promoters and appropriate trafficking of the PTGS agents into the diffuse or specific microenvironments in which the target RNA resides inside the cell (e.g., knowledge of target RNA zip codes and the capacity to integrate this into the PTGS strategy). The PTGS agent must be stable, resist nucleases, and have a long cellular lifetime in the appropriate cellular compartment. The accessibility of the target mRNA must be rigorously determined if any successful target suppression is to occur. Regions of the target that present large, stable, single-stranded annealing platforms appear to be optimal, but these sites are typically rare in any target RNA. The PTGS agent must be able to appropriately sample necessary conformational transitions to achieve its activity, an issue which is especially challenging for an Rz and especially when the Rz is embedded in a chimeric RNA. For an Rz or RNAi the binding to the target must be sufficient but not too tight in order to achieve maximum specificity and to allow product release that is necessary to support enzymatic turnover. In aggregate, these variables create rational engineering and experimental challenges which must be embraced simultaneously to achieve efficacious PTGS agents for candidate therapeutics. This multivariable problem is a major reason why the entry of PTGS into the therapeutic landscape for human disease has been so slow.

## 3. Strategies and Approaches for PTGS Therapy

There are several types of therapeutic strategies that might be used for PTGS by any technological modality. The choice may depend upon whether the disease process is genetic in origin and whether the target mRNA or viral RNA codes for a normal or mutant protein. These strategies are (1) mutation-independent or knockdown, (2) RNA repair, (3) mutation-directed, and (4) combined therapy. By example, we will discuss here the different types of strategies as they might be applied to human retinal (or ocular) diseases, which is this labs venue of interest. The generic knockdown approach may also be used to suppress wild-type mRNA expression [137].

### 3.1. Knockdown Therapeutic Approach

 The mutation-independent or knockdown (KD) approach is the most straightforward. This strategy is used to suppress or knock down a target mRNA and its cognate protein. The target mRNA may be overexpressed from wild-type (WT) genes in particular clinical conditions or may be expressed in normal amounts but a therapeutic benefit can be envisioned from target suppression, or the target could be a viral RNA essential to a viral life cycle. KD may also be used as a component of combined PTGS therapy for genetic diseases (see below). The initial goal with KD is to identify the single most accessible site(s) of the WT target mRNA or viral RNA. Once this site(s) is identified then PTGS agents (AS, Rz, and RNAi) can be designed to anneal at these regions and promote target RNA knockdown within the live cell. AS ODNs are typically transfected into cells of a particular type, where the target is expressed and where the disease process is manifest. Rzs or siRNAs RNAs may also be directly transfected into cells. More commonly, Rzs, shRNAs, or miRNAs are transcribed from plasmid or vector constructs by RNA polymerases (Pol-II or Pol-III) within the cell harboring the target RNA. Expression constructs for the PTGS agent are delivered into cells by transfection agents, viruses, or nanoparticle systems (synthetic viruses).

### 3.2. Gene Therapies for Dominant Mutations

 The molecular genetics of inherited retinal degenerations is very well developed and provides a suitable example. Many mutations in human genes that are expressed in the eye cause autosomal dominant disease patterns. Autosomal dominant hereditary retinal and macular degenerations are caused by mutations in genes expressed in specific cell types of the human retina [138, 139]. At least 204 retinal disease genes have been mapped, and 161 of these genes are cloned in part due to highly effective candidate gene approaches and the Human Genome Project and a database is available (RetNet, [140–142]). However, the RetNet database of disease genes underestimates the gene therapy challenge because multiple disease-causing mutations are commonly found in any given gene. The number of mutations can extend into the hundreds. Relevant examples are the many (>120) human rod opsin gene (*RHO*) mutations identified, since the P23H mutation was found causally associated with *adRP. *Opsin mutations are estimated to be responsible for 25%–30% of *all* cases of *ad*RP [105, 106, 138]. Mutations in the *RHO *gene cause *ad*RP, autosomal recessive RP, *ad *congenital stationary night blindness, and retinitis punctata albescens. The *RHO *gene, therefore, offers a robust *model* to investigate the extent to which PTGS therapies can be broadly applied as human gene therapies for *ad *retinal degenerations. The VMD2 or *Best-1* gene is another robust example. VMD2 is mutated in Best's vitelliform dystrophy and adult foveovitelliform dystrophy and has been found to harbor at least a hundred mutations, with almost all of these being dominant in nature. One can expect the general trend that the number of mutations identified increases with time after identification of the disease gene. 

#### 3.2.1. Mutation-Independent or Allele-Independent Approach

 The mutation independent (MI) or KD approach in its stand alone format may be sufficient to suppress the disease process in a dominant genetic disease provided that no haploinsufficiency results. In a dominant hereditary condition, one can expect approximately 50% WT and 50% mutant protein expression from the two alleles. Often, in the normal case (no mutations), the WT protein is expressed in excess over that needed for cellular functions. In the autosomal dominant condition, the WT protein is already reduced by approximately 50%. This may already be insufficient to support cellular metabolism in the absence of the mutant protein (haploinsufficiency). In an MI or KD approach, the best PTGS agent is identified to target the most accessible site in the target mRNA to achieve the greatest degree of target mRNA/protein suppression. In this strategy, there is no specificity for the mutant mRNA versus the WT mRNA. Knockdown of mutant protein expression is expected to ameliorate the cellular toxicity that results from protein misfolding, or gain-of-function properties of the mutant protein, and thus relieve cellular stresses and permit longer cell vitality or normalization of cellular function. However, the MI or KD PTGS agent will also reduce WT protein expression below 50%, and this could promote haploinsufficiency and cell stresses and even cell death as a result. Thus, the MI or KD approach, as described above for WT targets, may possibly be used in autosomal dominant hereditary conditions, provided that the cell can resist haploinsufficiency due to further reduction of WT protein. If the relief of cellular stresses due to a highly toxic mutant protein can come about by relatively small reductions in mutant and WT protein, the impact of haploinsufficiency may not play as strong a role in cellular vitality.

#### 3.2.2. mRNA Repair

**Figure 6:**
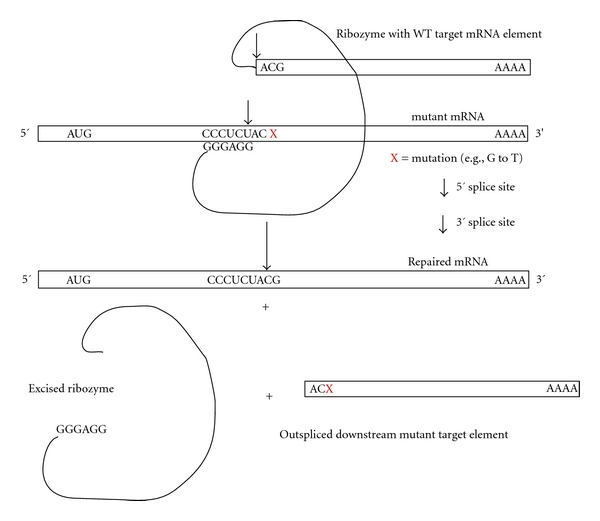
mRNA repair strategy for Dominant Mutations. The *trans-*splicing group I intron of Tetrahymena is engineered with an element that contains a WT mRNA sequence starting from just upstream of the mutation(s) in the target mRNA (labeled X). The Group I intron recognizes the region upstream of the target by way of complementary base pairing. It then cleaves the target using free guanosine as a nucleophile and then *trans-*splices a fresh downstream WT target mRNA element at the precise site of cleavage. All mutations in a given gene below the splice site could be treated with a single *trans-*splicing group I intron. One or several engineered group I introns could cover most mutations in a given human gene.

mRNA repair has been described with the use of the large trans-splicing Group I intron ribozyme of *Tetrahymena* that has been under development by the Sullenger and Haseloff labs [143–153] (Figure 6). The concept behind mRNA repair is that the guide sequence of the Group I Rz is engineered to anneal to a region of the target mRNA just upstream of the location of the mutation, cleave the RNA while using an available free guanosine as the nucleophile, release the downstream cleavage product, and finally trans-splice a normal 3′ exon onto the 3′ end of the upstream element of the target. All mutations in a target gene downstream of the cleavage splice site can thus be repaired. This makes mRNA repair an MI strategy. The engineered *Tetrahymena* Rz is actually a chimeric RNA and contains the Rz sequences, the guide sequence which is antisense to the target mRNA region, and an appended sequence which is the WT version of the target mRNA from just upstream of the site of mutation(s). The goal of mRNA repair is to cleave the target mRNA just above the site(s) of mutation and to splice onto the 3′ cleavage end an in-register copy of the downstream component of the WT mRNA. The ribozyme splices itself out during this process. In this two-step process a WT mRNA is produced. This strategy could be used to repair all mutations downstream of the site of targeting. Therefore, just a few sites of targeting might be used to repair most or all known mutations in a given disease gene. A major disadvantage of this approach is that only a short guide sequence in the Rz (6 nt) is used to recognize the target RNA. This has resulted in lack of specificity regarding off-target mRNAs. Recently, the antisense region was extended to improve specificity against the intended target, and other elements of the Rz were optimized to generate better efficiency of trans-splicing. As for other PTGS strategies, the site of targeted annealing for *trans-*splicing has been found to be substantially affected by the secondary and tertiary structure of the mRNA in mammalian cells. Therefore, to be able to handle all or most mutations in a given gene, several accessible sites in the target will generally be necessary or a single accessible upstream target may be sufficient. While this specific MI approach by mRNA repair is more complex relative to other modalities (e.g., hhRz, RNAi), RNA repair still has potential to become clinically useful [147, 154]. One limit is that multiple agents will likely need to be developed to handle sets of mutations in a given gene, unless an upstream accessible region can be used for all mutations in a given gene. Another important issue is that the engineered Group I intron is spent for each mRNA that is repaired. There is no target turnover as one would have with either Rz or RNAi agents operating as Michaelis-Menten PTGS agents. Once the RNA repair enzyme operates on a single target that ribozyme no longer has a 3′ WT region to append to a subsequent target.

#### 3.2.3. Mutation-Directed Strategies

Mutation-directed (MD) therapeutic strategies target only the mutant mRNA with the intent of leaving the WT mRNA intact. MD PTGS agents have been shown to have therapeutic potential to stably rescue photoreceptors from toxic mutant opsin protein expression manifest in a transgenic *ad*RP rat model, albeit only a small fraction of mutant mRNA was suppressed [155, 156]. First, we detail the specific uses of a MD strategy, and then, we will present the advantages and substantial disadvantages which we expect will limit its use in gene therapy. There are two means by which a MD strategy might be realized, which depend upon the nature of the mutation and the nature of the PTGS technology utilized. In a mutation-specific strategy (MSpe) PTGS agents selectively inhibit *mutant* genes by targeting *only* mutant mRNA for cleavage. MSpe PTGS can easily be embraced by hhRz technology given the high degree of specificity of cleavage of the target mRNA at NUH↓ sites. To be susceptible to a MSpe strategy, the mutation in the gene must create a *new* cleavage site that is not present in the WT mRNA. For example, consider the human *RHO* mutation G51V (GGC → GUC↓). The mutation at the gene level is a transversion converting a G to a T residue (or U for RNA). This coding region mutation converts a glycine codon to a valine codon and generates a new hhRz cleavage site (GUC↓). This coding mutation results in a mutant protein, when expressed with WT protein, that leads to photoreceptor stress and ultimately apoptotic cell death in *ad*RP [157–161]. The mutant protein could inhibit appropriate expression or trafficking of the WT protein (dominant negative mutation). It is possible that this specific mutation leads to a protein that is unstable in that it misfolds, is targeted for ubiquitination, and is then degraded by the protesome. Such a result in general could lead to haploinsufficiency as the assumed 50% of WT gene product that is made stably by the cell in a dominant hereditary condition may be insufficient to build the necessary multiprotein structure, create sufficient enzyme activity, or maintain the capacity of a signaling pathway. On the other hand, this mutation could promote a gain of function. Gain of function mutations could act at many levels. Such a mutation could create in the protein nonfunctional misfolded states that are not processed in large part for degradation, but rather become trapped in the endoplasmic reticulum, where they can accumulate and activate the unfolded protein response to exert toxicity and promote apoptosis [162–171]. On the other hand, the mutant protein might fold normally but mistraffic to the wrong compartment in the cell and exert signaling events that are a gain of function that is toxic to the cell (e.g., [172]). The mutant protein may fold and traffic correctly but have some intrinsic instability that results in aberrant interactions with other proteins or aberrant signaling events that are also gains of function (e.g., [173]). Or, the mutant protein, in its interactions with other macromolecular components or the WT protein itself, affects the processing, trafficking, structure building and functional expression levels of the WT protein and thus have a dominant negative influence [174–176]. The possibility of the mutant protein creating a haploinsufficiency, gain of function, or dominant negative effects has substantial impact on PTGS strategies. Haploinsufficiency is treatable with a WT allele. Dominant negative effects might be treatable with a WT allele but may also require mutant protein knockdown. Gain of function effects certainly requires mutant protein suppression. It is appropriate to consider potential therapeutics in terms of whether they require WT protein reconstitution, or alterations in the relative ratio of WT to mutant mRNAs and proteins. 

Returning to the RNA targets, the WT mRNA triplet GGC does not represent one of the classical NUH↓ sites for the hhRz, but the new GUC↓ motif is not only a classical triplet, but is also one of the two naturally occurring NUH↓ sites (GUC↓, GUA↓) with high intrinsic cleavage rates. The mutation creates a new hhRz cleavage site in the mutant mRNA, while the WT mRNA has no cleavage site at the same position. This is the *necessary* and *sufficient* condition for use of the MSpe strategy. Such a mutation creates opportunity to design an MSpe hhRz intended to cleave *only *the mutant mRNA while leaving the WT mRNA intact, because it lacks the new NUH↓ site [177, 178]. The mutation could occur at any position in the gene (5′UT, coding, 3′UT) and still allow an MSpe strategy so long as a new NUH↓ cleavage site is created. Such a stand-alone strategy only makes sense for autosomal dominant mutations. On face value, the MSpe strategy seems ideal given that it allows a specific attack of a PTGS agent only on the mutant mRNA to suppress the mutant disease protein. However, there are a number of substantial limitations to the MSpe strategy. First, the MSpe strategy would only be indicated when there is a toxic gain of function of the mutant protein in the cell in which it is expressed. Second, only a fraction of mutations that occur in a given disease gene would create *new *hhRz cleavage NUH↓ motifs required for MSpe design. For example, of the 124 human rod opsin *a*dRP mutations that we have tabulated relatively few (~21%) create new NUH↓ cleavage motifs for hhRzs. Similarly, of the 108 human VMD2 mutations that we have tabulated, only 15% create new NUH↓ cleavage motifs for hhRzs. Third, there is variation in the intrinsic rate of cleavage of NUH↓ motifs and some of these (e.g., AUA↓) have cleavage rates up to several orders of magnitude slower than those that occur in nature (GUC↓, and GUA↓) [43]. Fourth, even though the MSpe hhRz would be designed to cleave the new NUH↓ site created by mutation, most of the two antisense flank regions will precisely anneal to the WT target mRNA; this could result in a substantial antisense effect against the WT mRNA and contribute to an already preexisting haploinsufficiency effect due to the dominant mutation [178]. These factors alone would strongly limit the overall applicability of the MSpe approach for *ad *mutations in any gene except those that generate new *robust* hhRz cleavage sites. Fifth, random single-nucleotide mutations which constitute the bulk of human mutations are expected to mostly reside in regions of dense secondary structure, and be largely inaccessible for targeting. The expected lack of accessibility in the target mRNA around sites of most mutations is likely to be a single major factor that limits development of MSpe hhRz strategies. Sixth, each MSpe mutation requires an independent discovery and drug development process. The practical costs of such an effort are prohibitive. 

**Figure 7:**
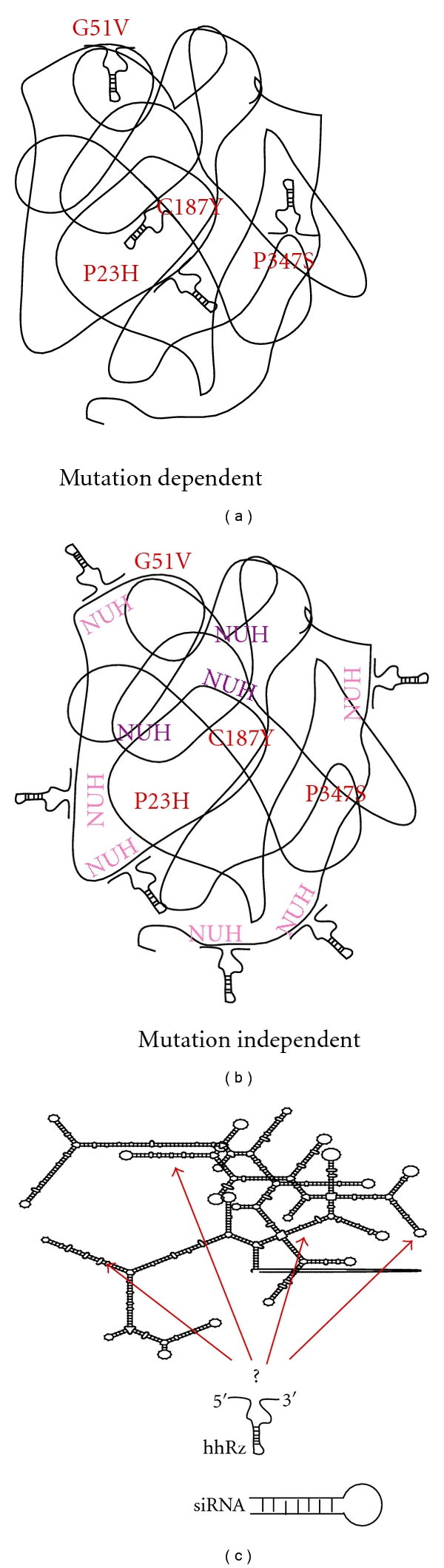
Comparison of mutation independent and mutation dependent strategies to PTGS therapy. The schematic representation shows two folded mRNAs, one in which a MD strategy is being used to attack discrete mutations which obligate the site of attack to regions that are likely to be buried, and the other is an MI strategy, where the best (most accessible) NUH↓ (lavender) or RNAi cleavage site is sought for use. This challenge applies to hhRz or RNAi type therapeutics.

A mutation-selective strategy (MSel) expands upon the limitations of the MSpe strategy [155, 179]. MSel hhRzs are designed to cleave at active NUH↓ sites (e.g. GUC↓, GUA↓, GUU↓, UUC↓, CUC↓, and AUC↓) that are *adjacent* to or in the immediate vicinity of the mutant codon. However, these cleavage sites are also present in the WT mRNA. This limits specificity (hence mutation selective), as some cleavage of WT mRNA will likely occur, in addition to the antisense (AS) effect on the WT mRNA that is expected to result from hhRz annealing [178]. The MSel rationale for development of an hhRz PTGS agent is that perfect hhRz annealing to mutant mRNA will lead to its selective cleavage, while mismatches between the hhRz and WT mRNA at N_1_, N_2_ or N_3_ in the sequence N_1_N_2_(NUH↓)N_3_ (H does not base pair to hhRz) will impair the cleavage rate for the WT mRNA (≥500-fold for an N_3_ mismatch = *Strong MSel*; ≤10-fold for an N_1_ or N_2_ mismatch = *Weak MSel*) due to an expected hhRz structural perturbation [47, 180]. Strong MSel hhRzs can target ~9% of opsin *ad*RP mutations, so we group them into a strong MD strategy (therapeutic potential for ~24% of mutants). All of the other disadvantages seen with the MSpe approach are also expected for the MSel approach, the most significant being target mRNA structure which will severely limit annealing at most sites of human mutation. In all MD strategies, the Rz is *obligated* to anneal to local primary sequence around the site of a random *human* mutation associated with an NUH↓ motif. Most single nt *ad *mutations in any mRNA will predictably localize to hybridized secondary structure which is expected to limit or frankly block Rz annealing at the vast majority of potential cleavage sites (see Figures 5 and 7). For example, attempts to develop mutation specific or selective ribozymes to the human rod opsin P347S mutant mRNA have failed *in vitro* [181].

All MD strategies of PTGS for genetic diseases require the design of the agent for a specific mutation or set of mutations (mRNA repair). The challenge to successfully build an efficacious PTGS agent has many pitfalls. This becomes greatly compounded when the design has to be repeated many times for a given disease gene, for example for those mutations where a strong MD strategy might possibly be feasible. The design and testing of a single PTGS agent requires extensive time, effort, and great expense when extending the development through the preclinical animal testing phase. And even if some such MD agents could be achieved, they likely would have varying efficacy to treat different mutations in a given gene. Yet, the development of such agents as drugs for orphan genetic diseases is clearly indicated for those suffering globally with such diseases. This need must ultimately be balanced by the fact that many human disease genes have substantial allelic heterogeneity or mutational diversity. Mutation frequency can vary from common with founder effects (e.g., P23H in human *RHO* gene) to rare (e.g., K296M in rod rhodopsin, [182]), where only a single family pedigree with two affected individuals has been identified globally to date. It is difficult to anticipate that rare genetic mutations would be targeted by a unique therapy that moves up through clinical approval and the many hundreds of millions of dollars that are needed to realize an effective and safe new drug. Research and development costs for PTGS gene therapy will be colossal if testing of many designs is needed to achieve optimized MD constructs for *each* mutant mRNA. Rather, what is rational to expect is that a single PTGS therapy directed to a single human disease gene might eventually come to fruition. While KD or MI PTGS therapy embraces a critical aspect of such an approach (one therapy for all/most dominant disease mutations in a single gene) the potential limitation of haploinsufficiency is already prompting development of combined therapeutic strategies.

### 3.3. Combined PTGS Therapeutic Strategies

A major advantage of the KD or MI strategy for autosomal dominant retinal or eye diseases is that the *best* hhRz or other PTGS agent can be sought to cleave the mutant (and WT) mRNA at the most accessible site and that a single KD agent can be used to cleave many or *all *mutations so long as the binding or NUH↓ cleavage motifs are not affected by mutation (probability <0.008). The KD strategy avoids repetitive and expensive R&D for each new mutant as per the MD strategy. One KD agent could provide therapy for all or most mutations in each disease allele. We, and others, have developed KD hhRzs that cut full-length mutant *human* rod opsin mRNA and could be used to target all currently known opsin *ad*RP mutations [128, 183–189]. Since the most optimal (accessible) cleavage site for the WT (and mutant) mRNAs is sought for attack, the critical limiting variable in development of PTGS agents, target RNA structure, is immediately embraced by this strategy. The single and substantial disadvantage of the KD strategy is that both mutant and WT mRNAs are expected to be equivalently cleaved by the PTGS agent. The cellular phenotypic outcome of expected equivalent knockdown of both WT and mutant mRNAs and proteins will depend critically upon the cell in which the gene is expressed, the function of the protein, and the resultant levels of expression induced by the MI PTGS agent. Let us consider *RHO *as a target of KD PTGS. Rod opsin is expressed in abundance in rod photoreceptors and is the visual pigment that subserves human scotopic vision. There is a plethora of biochemical, biophysical, cell biological, and genetic data on rhodopsin from over four decades of research. WT rod rhodopsin is expressed in great excess in photoreceptors to the levels over 2 × 10^8^ copies/cell. Essentially, all of the apoprotein is trafficked to the outer segment, where over 98% is localized to topologically isolated disk membranes and under 2% is localized in the plasma membrane. The human rod photoreceptor has the capacity to detect and respond to the absorption of single photons of appropriate energy, in part due to an extremely low level of electrophysiological noise in darkness and a high gain biochemical amplification pathway in light. Nevertheless, the dynamic physiological range of a human rod photoreceptor saturates upon approximately 200 photon absorptions [190]. Therefore, there are 99.9999% spare rhodopsin receptors in the rod photoreceptor to guarantee quantum catch when photon density is extremely low (dim starlight). There is substantial evidence that the rod photoreceptor autoregulates the amount of opsin that is expressed in order to maintain a constant daily absorption of photons in a process called photostasis [191]. Rhodopsin itself appears to be the sensor that drives this transcriptional regulation pathway. In rodents kept in dim light, the levels of opsin expression increase, and the outer segment length increases with no apparent change in diameter. In increasingly higher levels of light, the level of opsin expression decreases proportionally, and the outer segment shrinks in length. At sufficiently high levels of light, there is light damage and cell death. How much WT opsin is necessary to maintain the vitality and ideally the function of the rod photoreceptor? This is a systems biology question of critical relevance to the KD PTGS approach to gene therapy of opsin-based *ad*RP. We do not yet know the full answer, but studies have pointed to an understanding of gross limits on the range of normal rod opsin expression that are needed to maintain the structure and physiological vitality of the rod photoreceptor. There is substantial evidence that rhodopsin is in *at least* 50% excess for long-term structural maintenance and survival. A recessive human mutation, E249ter, causes 50% loss of WT rhodopsin but is phenotypically *silent* in the carrier state [192–194]. The heterozygous rod opsin mouse (50% rhodopsin/rod) is similar to human E249ter carriers in that very slow, if any, retinal degeneration occurs over 90–120 days [195–197]. With only 50% of normal WT rhodopsin being present, the outer segment lengths have shrunk to approximately 50% of their normal length while apparently maintaining their diameters. When rhodopsin levels decrease to 25% of normal in rats exposed to moderate light intensity, *many* rods maintain vitality with shorter outer segments, but many rods also die [191]. One might, therefore, hypothesize that a significant reduction (between 50%–75%) of WT rod rhodopsin (rod sensitivity reduction by −0.3 to −0.6 log) would not cause *rapid *retinal degeneration in mammals. Efficacious PTGS knockdown (knockout is unlikely) of 50% of *total *opsin protein would leave 25% WT and 25% mutant in *ad*RP rods. With severe mutants (e.g., C187Y), the benefits of reducing toxic gain of function mutant protein are expected to offset partial loss of WT opsin. However, WT opsin levels must be maintained at around 50% to permit rod survival *in mouse* [195, 196]. When the E249ter mutation is homozygous and no WT opsin is synthesized, affected patients have early onset autosomal recessive RP [192]. The mouse opsin knockout has a rapid retinal degeneration. Opsin expression is essential for stable elaboration of an outer segment and the formation of the phototransduction apparatus. A single WT allele slows degeneration in the presence of a single mutant opsin allele in mice [198]. This suggests that the WT allele is protective, at least under certain constraints. On the other hand there is a distinct limit on overexpression of the WT opsin protein. Tan et al. [199] showed in murine transgenic models that overexpression of WT rod opsin in rod photoreceptors beyond 125% of normal levels promotes retinal degeneration. Thus, it would appear that the tolerable limits of under and overexpression of WT rod opsin in the mammalian rod photoreceptor likely range from between 25% and 125%. This broad range indicates that the photoreceptor as a system is highly tolerant or capable of major fluctuations of one of its critical functional proteins. Many other phototransduction, structural, and metabolic proteins are expected to shift their expression levels in concert with opsin, as they are cotranscriptionally regulated. This may have substantial functional implications for cellular adaptations such as photostasis. If WT rhodopsin levels at 25% and above exceed a threshold supportive of rod photoreceptor vitality with an outer segment, then a relatively efficacious PTGS agent that knocks down 50% of total opsin protein (WT and mutant) would be expected to permit rod photoreceptor survival if the WT fraction was the only component for consideration. However, the impact of the mutant protein could be a toxic gain of function modality for the cell. The level of knockdown of the *mutant* fraction that is necessary to support vitality of the rod photoreceptor will likely prove to be dependent upon the nature of the mutation and the levels of photoreceptor systems biology that are impacted by any gain of function toxicity. At present, we can only anticipate that knockdown of mutant and WT protein levels will vary depending upon the PTGS agent that is used and its expression level in the appropriate cell type. It is prudent to expect that there will be a dynamic range of potential therapeutic outcomes from a single PTGS agent in a given cellular system. Any therapeutic rationale must embrace the intrinsic dynamic range of WT protein expression, varying toxicity of the mutant protein, varying therapeutic efficacy of the PTGS agent itself, and a means of transcriptionally regulating the PTGS agent both to tune the therapeutic effects or modulate against potential deleterious effects. These issues which tap into retinal systems biology make PTGS therapy a difficult but likely attainable goal in the road ahead.

#### 3.3.1. Combined Knockdown: Reconstitution Therapy

 The combined knockdown: reconstitution therapy (CKDRT) embraces both the knockdown potential of the PTGS agent and the protective effect of the WT allele*. *In CKDRT, both the native WT mRNA and the mutant mRNA are targeted for therapeutic KD PTGS attack, but the WT mRNA levels are reconstituted through expression of an engineered allele that transcribes a WT mRNA that is resistant or hardened to cleavage. Montgomery and Dietz [200] first reported that KD hhRzs embedded in an antisense sequence were able to efficiently cleave fibrillin-1 mRNA (disease gene in *ad* Marfan's syndrome). They suggested a general approach in treating a variety of *ad* genetic diseases by a knockdown hhRz/AS to suppress both mutant and WT mRNAs in association with a WT reconstitution construct, altered with respect to codon degeneracy, to reconstitute WT expression to appropriate levels in order to prevent intrinsic or therapeutic haploinsufficiency. It is this strategy that we call CKDRT. Later in the same year, Millington-Ward et al. [183] reported that ribozymes against opsin and peripherin mRNAs could potentially be used in a CKDRT strategy as a general approach for therapies of *ad *genetic diseases. CKDRT has also been applied to *ad α*-1 antitrypsin deficiency of liver [201, 202]. There is increasing utility of the CKDRT approach combined with a decreased frequency of reports on design of MD PTGS agents [128, 183–185, 187, 188, 203–206]. This is predictable given the severe constraints of the MD approach as presented above. Nevertheless, while CKDRT may be a suitable approach to clinical gene therapy, for an autosomal dominant disease, there remains many significant scientific hurdles to be overcome, some of which we will present here. The first goal beyond development of a potent KD or MI PTGS agent is to achieve a functional allelic variant WT (*a*WT) mRNA with full potential to translate sufficient levels of WT protein given normal levels of transcription.

#### 3.3.2. Design of Allelic Variant WT Expression Constructs

A critical component of the CKDRT approach is that an *a*WT variant of the WT gene or cDNA must be engineered to express a processed mRNA that is resistant or hardened to cleavage by the specific KD or MI PTGS agent. We consider how target mRNA resistance can be engineered when the PTGS strategies use hhRz or shRNA modalities. The design of a cleavage-resistant mRNA for reconstituting WT protein expression can be simple or complex, depending upon the location and nature of the cleavage site in the mRNA relative to the reading frame of the protein. We will first consider the development of an hhRz resistant *a*WT variant for three attack sites in a given mRNA to indicate the potential complexities in *a*WT variant design. We will use the rod opsin (*RHO*) mRNA as a model in part, because there is a crystal structure available for the WT rhodopsin protein that is encoded by this mRNA [207]. The protein crystal structure can guide decision making in complex *a*WT variant gene design, when the region of attack is in the protein-coding region and the cleavage site occurs within rather than at the end of a discrete codon. Consideration of WT protein structure is essential to maintain the WT structure/function and phenotype within the cell in which the *a*WT construct would be expressed, especially in the specific case when the *a*WT construct cannot be made silently with respect to the protein reading frame. We will assume three sites for hhRz attack with one in the 5′UT and the remaining two in the coding region of the mRNA (Table 3). Let us further assume that these three sites have equivalent and high levels of accessibility such that they would be sensible regions for PTGS attack by a KD, MI hhRzs, or shRNAs. The 5′UT and 3′UT regions of the processed mRNA are the easiest regions for design of a *a*WT construct because maintaining appropriate amino acid protein coding is not a variable.

***a*WT Construct Design for hhRz PTGS Agents**

**Figure 8:**
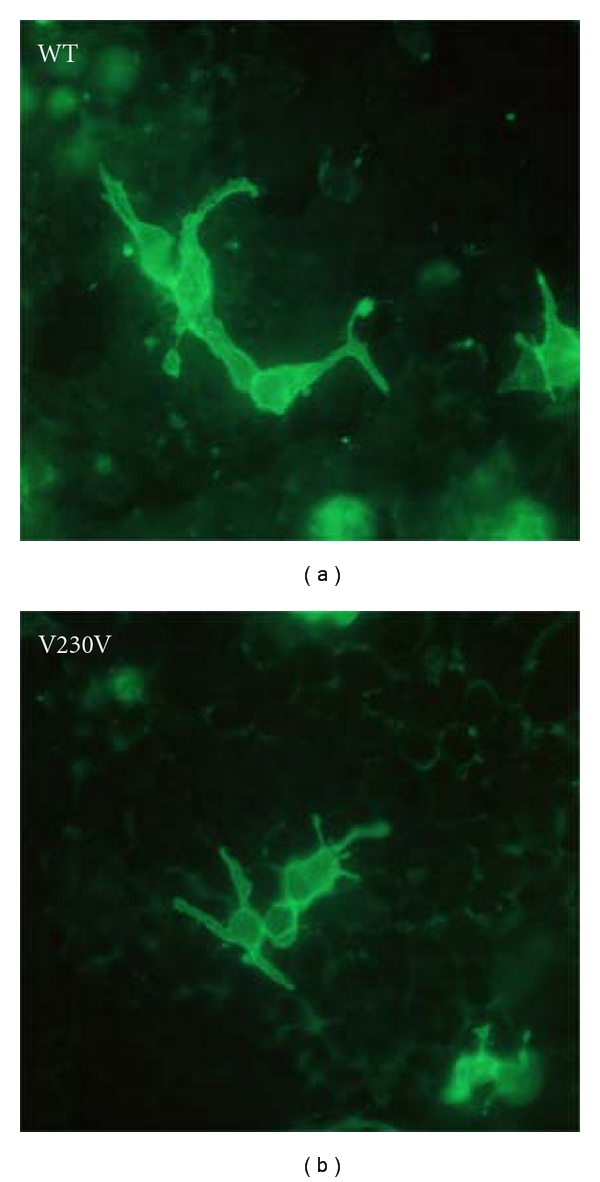
Allelic variant human opsin construct. The hhRz cleavage site at V230 (GUC↓) was mutated by site-specific mutagenesis to the degenerate human valine codon GUG. The V230V human opsin cDNA in a CMV expression vector (pCDNA3) was expressed in HEK293S cells along with control human WT opsin CMV expression vector. Immunocytochemistry with 1D4 opsin monoclonal and an FITC-labeled secondary antibody was conducted. Abundant human WT opsin expression and cell surface trafficking was noted in cells expressing WT (a) or V230V *a*WT proteins (b).

**Figure 9:**
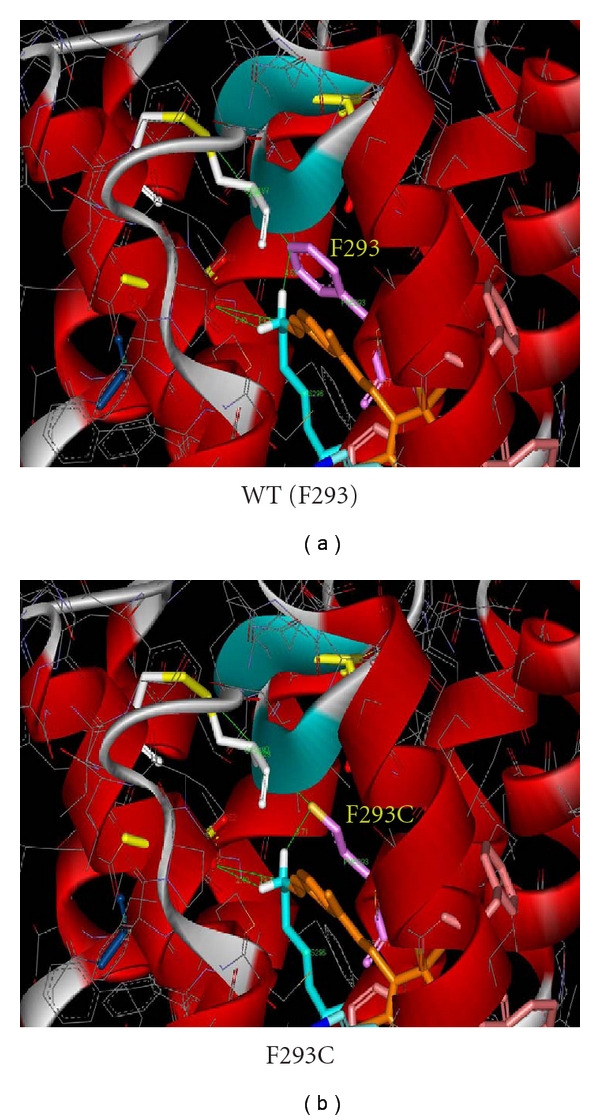
Crystal structure analysis of allelic variant constructs. We analyzed the location of the F293C mutation in the bovine rod opsin crystal structure (1F88.pdb). WT protein (a). The image shows the cutout region around the protonated Schiff base linkage of 11-*cis-*retinal (orange) to K296 (blue sidechain). F293 is lavender in color. The disulfide bond between C110 and C187 is in yellow. F293 is within 5 Å of the Schiff base and within 10 Å of the disulfide bond. F293C Mutation (b). The C293 sidechain is lavender in color with a yellow tip indicating a free sulfhydryl group (-SH). The SH group is within 5 Å of the Schiff base and 10 Å of the disulfide bond.

For a CUC↓ hhRz attack site in the 5′UT, it is relatively simple to obviate the proven efficacious hhRz cleavage at this site by a single nt change from CUC↓ to CUG (Table 3). An hhRz cleavage site is NUH↓ where N is any nt and H is any nt except G. CUG is representative of any NUG site (GUG, CUG, AUG, and UUG) that cannot be cleaved by a hhRz. Hence, any chosen NUH↓ site in an accessible region of the 5′UT (or 3′UT) could be converted to an NUG site which cannot be cleaved to generate an *a*WT construct. If the hhRz targeting this CUC↓ site has been shown to exert all of its KD on the basis of RNA catalysis, with the loss of all KD occurring through catalytic enzyme core mutations, then this simple mutation creates a sufficient *a*WT construct. One also wants to avoid the potential impact of significant antisense effects of the chosen PTGS hhRz agent on the *a*WT mRNA. Next, we consider two hypothetical accessible hhRz cleavage sites in the coding region of the opsin mRNA. First, let us consider the site at V230 which is encoded by a GUC↓ triplet. *In vitro* hhRzs were designed that were able to cleave at this site in the human *RHO *mRNA [185]. A single nt transversion leads to a GUG triplet which is no longer cleavable by the targeting hhRz. Moreover, GUG still codes for V230 due to degeneracy. *In vitro *hhRzs that successfully cleaved targets containing the GUC↓ site failed to cleave the *a*WT variant RNA containing the GUG triplet [185]. We engineered an *a*WT V230V (GUG) variant cDNA by site-specific mutagenesis and put this construct under the control of a strong CMV promoter in a cellular expression plasmid. When we expressed this *a*WT V230V (GUG) cDNA in HEK293S cells, we found, qualitatively, that the expression and cellular distribution of WT protein was not different from otherwise equivalent V230 GUC↓ WT expression construct by immunocytochemistry (Figure 8). Assuming that a hhRz exerts full catalysis at this site without substantial pure AS effects, an *a*WT with potential for success has then been designed. Design of an *a*WT construct at the second coding site (F93) demonstrates the complexity that can arise, however, because an NUH↓ cleavage site within the coding region may not obey the serial order of protein coding triplets but rather overlap them. The assumed accessible targeted triplet GUU↓ overlaps two codons of human *RHO* mRNA at A292 and F293 (Table 3). To make an *a*WT mRNA that is resistant to hhRz attack the conversion of GUU↓ to noncleavable GUG results in a F293C (phenylalanine to cysteine) mutation in the opsin polypeptide. Moreover, the location of this mutation at the protein level is one helical turn away from the side chain of K296, which is the site of covalent attachment of 11-*cis-*retinal to the opsin apoprotein. The replacement of a phenylalanine with a cysteine side chain in such a critical location in the protein as an *a*WT construct must be seriously considered. It is unclear whether the mutation represents an identified allelic variant WT or whether it is truly a mutation with a potential phenotypic effect on protein folding, function, or even potential toxicity (gain of function). The impact of such a mutation may be appreciated at a structural biological level if there is an available crystal structure. Also, expression of the mutant and normal proteins will be needed to compare functional profiles, if adequate assays are available (e.g. [208, 209]). A cellular expression to test for cell localization of the mutant versus WT protein clearly is indicated to insure that the variant protein has appropriate WT trafficking phenotype. Tests for expressed mutant protein structure-function relative to the native WT protein are also needed. An opsin F293C mutation replaces a hydrophobic aromatic ring sidechain with a polar and potentially reactive linear sidechain. The location of the normal and mutant sidechains in the rhodopsin crystal structure is shown (Figure 9). While the length of the mutant sidechain is similar to the native phenylalanine sidechain, the potentially reactive cysteine sulfhydryl group is approximately 4 Å from the Schiff base of K296 and 9 Å from the C187 or C110 sidechains. A cysteine sidechain residue in this position could alter the local environment important for 11-*cis-*retinal docking and covalent ligation, or it could impair or intrude upon disulfide bond formation between C110 and C187, which is essential to the tertiary structure of rhodopsin [210]. We found no biochemical structure function studies in rhodopsin that reported on mutations at F293, so it is unknown how well they might be tolerated, and coincident biochemical or biophysical structure function studies at the protein level could be important to assure that the allelic variant protein indeed has WT characteristics. While such a mutant protein that behaves like WT may be useful as an *a*WT variant, rigorous experimental proof will be necessary whenever development of an hhRz resistant mRNA requires the development of such a potential *a*WT variant. Clearly, if equivalently accessible regions present NUH↓ sites for targeting, where it would be easier to construct an *a*WT variant, it is prudent to consider further development of CKDRT PTGS agents for such sites.

***a*WT Construct Design for RNA_i_ PTGS Agents**

The manner in which an *a*WT- or RNAi-resistant target needs to be designed is based strictly upon codon degeneracy within the coding region of the protein, with little apparent restriction elsewhere in the 5′UT and 3′UT regions, except for otherwise unknown protein-binding regions that might only be discovered empirically. For an *a*WT construct to be built for resistance of the mRNA to annealing and cleavage of charged RISC within the coding region of the protein, it is necessary to exploit codon degeneracy to preserve the amino acid sequence while substantially perturbing the binding energy of the RISC to the target. Ideally, one will want to preserve, to the best extent possible, the use of the human codon bias (or animal codon bias in proof-of-principle studies) in the selection of alternative coding triplets, whenever these present so as not to potentially impact WT protein expression levels. We demonstrate an example of such a design (Table 3). As for the design of an *a*WT variant for hhRz resistance, it is necessary to empirically test for resistance to knockdown of the *a*WT expression construct mRNA in cultured cells relative to the original WT expression construct. The RISC complex of the antisense strand can tolerate several mismatches at the 5′ and 3′ ends and still be capable of cleavage [75]. While the energetic rules of nearest neighbor RNA: RNA binding of the guide sequence *within *RISC to a potential target are not yet well established, it is probably worthwhile to use nearest neighbor calculations to minimize the binding energy of the charged RISC to the *a*WT mRNA while preserving amino acid coding.

## 4. Conclusions and Outlook

The development of a successful PTGS agent for therapy is one of the more difficult tasks in molecular medicine today and is a task that is well described by the term biocomplexity. This biocomplexity is underscored by the fact that currently there is only a single PTGS agent that is FDA-approved for human use despite decades of academic and corporate research. Here, we have presented an overview of currently used PTGS technologies, the critical biophysical variables that impact efficacy, and the strategies that may be used for genetic or nongenetic retinal diseases, where PTGS agents are likely to have future therapeutic impact. We anticipate that ribozymes have substantially greater therapeutic potential than RNAi, because they can likely be as potent, given a predetermined accessible region in the target mRNA, yet they are not as fraught with the promiscuous off-target effects and toxicity that continues to be demonstrated with RNAi (shRNA, and siRNA). We have presented an overriding strategy used in this lab for development of hhRzs as therapeutic agents. We have tried to present not only a base of knowledge to begin work along this path, but also a view of the pitfalls so that other investigators may find it easier to proceed down these investigative paths in the interests of the patients. 

There are several remaining issues that limit wide-scale development of RNA drugs [95]. First, one must have highly reliable and efficient tools to first solve the severe problem of identifying those rare regions of target accessibility for annealing of PTGS agents. Work in this lab has focused on this problem of target mRNA accessibility with the development of several HTS bioinformatics and experimental approaches ([211, 212], Taggart et al., *in preparation*). Work must be directed to efficient methods of searching for these rare sites when the target is presented in biologically complex mixtures such as the cell cytoplasm. Second, once such accessible regions are determined there will likely be a substantial number of PTGS agents that will need to be tested for cellular efficacy and toxicity. HTS approaches to screening for cellular knockdown and toxicity by Rzs or other PTGS agents are needed to be able to quantitatively assess and rank order both efficacy and toxicity of sets of PTGS agents targeting given accessible regions (Yau and Sullivan, submitted; Kolniak and Sullivan, 2011; Butler et al., *in preparation* [213–215]). Third, without colocalization with target mRNA, any PTGS agent will fail at efficacy. Better tools to rapidly identify cellular target RNA trafficking routes, destination zip codes, and the sequence or structural motifs that effect such localization are needed. The later motifs could then be integrated into the PTGS agent constructs as complex chimeric RNAs with therapeutic, protective, and trafficking domains. Fourth, while it is generally accepted that Rz kinetic performance *in vivo *against native structure target RNAs is 100–1000 fold less effective than *in vitro *when measured against short unstructured model substrates, it has been difficult to evaluate the robust kinetic performance of ribozymes in mammalian cells. Approaches must be developed to determine the kinetic performance and rate limiting step(s) of a given PTGS agent *in vivo*, which is paramount to rational improvements for higher efficacy, and to understand the factors that influence intracellular failures of PTGS agents. Recent development of similar approaches in yeast might be beneficial to guide the way for kinetic Rz analysis in mammalian cells [216]. The engineering of *in vivo *cellular reporter systems for both the target mRNA and the PTGS agent might also be useful (Yau and Sullivan, *submitted*). Fifth, HTS assays to quantitatively assess the cellular levels of target mRNA and protein which will need to move beyond the classical gel-based approaches which are slow, complex, and semiquantitative and have high variability. We have developed a quantitative robotic imaging platform that is able to measure target proteins and mRNA in transfected cells that are fixed and permeabilized in 96-well format [213, 215] Butler et al., *in preparation*. Sixth, the power of macromolecular RNA as a drug must embrace rational and computational design for structure/activity assessments. Computational and biophysical approaches to this problem are emerging but will still currently require a fairly compact therapeutic RNA design to utilize such tools. Seventh, and finally, more efficient means of preclinical analysis of PTGS agents in appropriate animal models of disease are needed. Intraocular injections and certainly subretinal injections are complex multivariable surgical procedures when done on human eyes, let alone small mouse eyes [217]. Assessing the actual area of transduction as a normalization parameter to either histological or electrophysiological assays of rescue is paramount. In addition, for RNA drugs with potential to translate to the human condition, the target mRNA that drives the disease process in the animal models should be a full-length human mRNA that recapitulates both the target and the disease that will exist in future human clinical trials. Even though the primary sequence may be homologous over regions of PTGS targeting among mammalian cognate mRNA targets, it is the secondary and tertiary structure of the mRNA that governs accessibility and hence efficacy [212]. With human copies of the target mRNA in the animal model, there is more confidence that preclinical efficacy has hope of similar human clinical translation. Currently, such animal models are rare [218]. This lab remains dedicated to the resolution of bottlenecks in RNA drug discovery and development of tools that will hasten the pace of development of efficacious and safe PTGS agents as candidate therapeutics for human retinal and macular degenerative diseases.

## 5. Materials and Methods

### 5.1. RNA Secondary Structure Prediction

 The secondary structure of full length *RHO *and *BEST-1 *mRNAs were subjected to analysis, using free energy minimization (RNA-Fold, MFold algorithm) [113], a Boltzmann-weighted sampling of all substructures (SFold algorithm) (not shown) [219–221], and local free energy analysis by OligoWalk [222] (not shown). RNA-fold was used with defaults to obtain MFE structures of the larger RNAs for Figure 5. MFold was used at 37°C with 10 kCal/mol window, for a maximum of 99 structures, and with a difference window of 3 bp. OligoWalk used the MFold output to obtain a LFE map along the mRNAs. A window of 15 nt, corresponding to symmetric 7/7 nt hhRz, was used to calculate the local free energy to break the target mRNA. Regions of low LFE (less negative or positive Δ*G*) indicate regions of low secondary structure or dynamic fluctuations.

### 5.2. Molecular Graphics

 The bovine rod rhodopsin crystal structure (1F88.pdb) was visualized and annotated with ViewerPro (vers. 4.2) software (Accelrys). Changes in amino acid sidechains were made with the same software. There was no effort to minimize the energy around the local site of amino acid mutation (F293C) so the location of the sidechain is approximate.

### 5.3. Site Specific Mutagenesis, Stable Opsin Cell Line Generation, and Immunocytochemistry

 These approaches and methods have been described in detail elsewhere [209].

## Figures and Tables

**Table 1:** Comparison of the properties of antisense, ribozyme, and RNA_i_.

Property	Antisense	Ribozyme	siRNA/shRNA/miRNA
Size	Small (15–20 nt)	Small (42–60 nt)	Small (19–22 nt)*
Crystal structure	No	Yes	Yes (RISC)
Mechanism of action	Known	Known	Known
Independence cell metabolism	No	Yes	No
Specificity	Moderate	High	Poor to moderate
Saturable	No	No	Yes
Cellular compartment	Cytoplasm	Nucleus/cytoplasm	Cytoplasm
Dependence on target structure	Yes	Yes	Yes
Proven *in vivo *	Yes	Yes	Yes

*On one strand engaged in the RISC complex after cellular processing.

**Table 2:** Methods to identify accessible sites in target RNAs.

Method	Type	Properties	References
MFold	IS	Algorithm finds minimal free energy (MFE) structure and set of lower energy structures. Display as pictorial structures or output as a single-stranded frequency map vector (probability estimator).	[14–17, 22, 95, 112, 113, 124, 125, 128, 211, 223, 224]
SFold	IS	Algorithm searches all of folding space and samples on basis of free energy and determines probability of access directly.	[128, 221]
OligoWalk	IS	Algorithm takes output from MFold (.CT file) and uses this to determine local target unfolding energy, ligand binding energy, and net energy.	[25, 222]
mppRNA	IS	Uses MFold, SFold, OligoWalk, and in-house processing model to predict net probability of access in a region and to rank order the outcomes based on several parameters.	[95, 113, 128, 136, 211, 221]
ODN: RNaseH	EX	Search combinatorial ODN library for those entries able to bind to target RNA on basis of RNaseH of RNA: DNA hybrid, followed by primer extension analysis. Gel-based and cumbersome.	[14, 15, 31, 123–125, 225–227]
ODN arrays	EX	AS ODN sequence overlapping arrays are tiled onto silicon surfaces. Labeled target RNA is bound under defined conditions. Target binding to regions of the array identifies accessible regions.	[18, 228, 229]
Rz library	EX	Rich combinatorial library of hhRz sequences was used to cleave target RNA. First strand cDNA primed by Oligo-dT was followed by 3′ dG tailing, followed by PCR with a downstream gene-specific primerv and a poly-dC allowed amplification and sequencing to determine cleavage sites.	[136, 230–233]
RT-ROL	EX	Uses probe for reverse transcription that has 3′ randomized region to screen for accessibility and constant region for PCR. Gene-specific upstream primers allow agarose gel-based mapping of accessible sites for antisense or ribozymes. Requires concurrent sequencing analysis for mapping.	[234]
RT-TDPCR	EX	Cleavage by AS or Rzs is followed by RT, 3′cDNA tailing, and then PCR using a tail-specific primer and a downstream gene-specific primer. Very sensitive.	[235]
cMARS	EX	Uses probe for reverse transcription that has 3′antisense to all NUH↓ hhRz cleavage sites, followed by randomized region to screen for accessibility and 5′ constant region for PCR. Gene-specific upstream primers allow agarose gel-based mapping of accessible hhRz cleavage sites and their relative accessibility.	[95, 211]
MAST	EX	ODN with upstream and downstream constant regions embracing region of randomized sequence. Constant regions clamped by annealing complements. ssDNA region of MAST tags probes RNA target attached to beads. Annealing followed by washing, probe displacement, PCR, and sequencing. Little capacity to discriminate signal from noise.	[236]
gsMAST	EX	Refined version of MAST in which the library is gene-specific or sequence-specific MAST tags against a target RNA are evaluated in competitive hybridization assay.	[95, 211]

Notes: cMARS: cDNA mapping of accessible ribozyme sites; EX: experimental (method); IS: *in silico* (method); gsMAST: gene-specific MAST; MAST: mRNA accessible site tagging; mppRNA: multiple parameter prediction of RNA accessibility; RT-ROL: reverse transcription with random ODN libraries; RT-TDPCR: reverse transcription, terminal transferase-dependent PCR.

**Table 3:** Construction of allelic variant genes for combined knockdown reconstitute therapies.

Allelic variants for HhRz Therapeutics	
5′ UT Target Site	

5′…CCUGAGUGGCUGAG**CUC**↓AGGCCUU…	(5′ UT target site CUC↓)
5′…CCUGAGUGGCUGAG**CUG** AGGCCUU…	(*a*WT variant mRNA, CUG cannot be cleaved)

Coding V230 region	
5′…CUC GUC UUC ACC **GUC**↓ AAG GAG GCC…3′	(Coding region GUC↓)
L226 V227 F228 T229 V230 K231 E232 A233	(Amino acid triplets)
5′…CUC GUC UUC ACC **GUG** AAG GAG GCC…3′	(*a*WT variant mRNA,
L226 V227 F228 T229 V230 K231 E232 A233	GUG cannot be cleaved)
Single letter amino acid codes are used.	

Coding F293 region	
5′…AUC CCA GCG UU↓C UUU GCC AAG AGC…3′	(Coding region GUU↓)
I290 P291 A292 F293 F294 A295 K296 S297	
GUU↓ cleavage site occurs *within *the F293 codon rather than cutting at the end of a codon.	
5′…AUC CCA GCG UGC UUU GCC AAG AGC…3′	(*a*F293C variant mRNA)
I290 P291 A292 C293 F294 A295 K296 S297	
It is unclear whether or not the F293C mutation is an allelic variant WT or has a protein phenotype.	

Allelic variant for RNAi therapeutics in F293 region	
5′…AUC CCA GCG UUC UUU GCC AAG AGC…3′	(Coding region RNAi site)
I290 P291 A292 F293 F294 A295 K296 S297	
5′…AUA CCC GCA UUU UUC GCG AAA AGG…3′	(*a*WT variant mRNA)
I290 P291 A292 F293 F294 A295 K296 S297	
*a*WT variant generated by codon degeneracy across the region of designed RNAi antisense annealing.	

## Abbreviations

*a*WT: Allelic variant wild type expression construct
AS: Antisense
*a*WT: Allelic variant wild type construct
*BEST-1*: Best macular dystrophy gene
CKDRT: Combined knockdown reconstitute therapy
dsRNA: Double stranded RNA
Δ*G*: Free energy (kCal/mol)
hhRz: Hammerhead ribozyme
hpRz: Hairpin ribozyme
HTS: High throughput screening
KD: Knockdown approach to therapy
MD: Mutation dependent approach to PTGS therapy
MFE: Minimum folding energy
MI: Mutation independent approach to PTGS therapy
miRNA: Micro-RNA
MSel: Mutation selective approach (under MD)
MSpe: Mutation specific approach (under MD)
nt: Nucleotide
ODN: Oligodeoxynucleotide
PAZ: Piwi Argonaut Zwille
PTGS: Post-transcriptional gene silencing
*RHO*: Rod opsin
RISC: RNA-inducing silencing complex
RNAi: RNA interference
Rz: Ribozyme
shRNA: Short hairpin RNA
siRNA: Short interfering RNA
WT: Wild type.
